# Synthesis and Cytotoxicity Studies of Novel NHC*-Gold(I) Complexes Derived from Lepidiline A

**DOI:** 10.3390/molecules23082031

**Published:** 2018-08-14

**Authors:** Danielle Curran, Oyinlola Dada, Helge Müller-Bunz, Matthias Rothemund, Goar Sánchez-Sanz, Rainer Schobert, Xiangming Zhu, Matthias Tacke

**Affiliations:** 1 School of Chemistry, University College Dublin, Belfield, Dublin 4, Ireland; danielle.curran@ucdconnect.ie (D.C.); oyinlola.dada@ucdconnect.ie (O.D.); helge.muellerbunz@ucd.ie (H.M.-B.); goar.sanchez@ichec.ie (G.S.-S.); xiangming.zhu@ucd.ie (X.Z.); 2 Organic Chemistry Laboratory, University of Bayreuth, Universitätsstr. 30, 95440 Bayreuth, Germany; bt303719@uni-bayreuth.de (M.R.); rainer.schobert@uni-bayreuth.de (R.S.); 3 Irish Centre of High-End Computing, Grand Canal Quay, Dublin 2, Ireland

**Keywords:** lepidiline A, *N*-heterocyclic carbene, gold anticancer drug, TrxR inhibition, MTT cytotoxicity assay, DFT calculations

## Abstract

Ten novel *N*-heterocyclic carbene gold(I) complexes derived from lepidiline A (1,3-dibenzyl-4,5-dimethylimidazolium chloride) are reported here with full characterisation and biological testing. (1,3-Dibenzyl-4,5-diphenylimidazol-2-ylidene)gold(I) chloride (NHC*-AuCl) (**1**) was modified by substituting the chloride for the following: cyanide (**2**), dithiocarbamates (**3**–**5**), *p*-mercaptobenzoate derivatives (**12**–**14**) and *N*-acetyl-l-cysteine derivatives (**15**–**17**). All complexes were synthesised in good yields of 57–78%. Complexes **2**, **12**, **13**, and **14** were further characterised by X-ray crystallography. Initial evaluation of the biological activity was conducted on all ten complexes against the multidrug resistant MCF-7^topo^ breast cancer, HCT-116^wt^, and p53 knockout mutant HCT-116^−/−^ colon carcinoma cell lines. Across the three cell lines tested, mainly single-digit micromolar IC_50_ values were observed. Nanomolar activity was exhibited on the MCF-7^topo^ cell line with **3** displaying an IC_50_ of 0.28 μM ± 0.03 μM. Complexes incorporating a Au–S bond resulted in higher cytotoxic activity when compared to complexes **1** and **2**. Theoretical calculations, carried out at the MN15/6–311++G(2df,p) computational level, show that NHC* is the more favourable ligand for Au(I)-Cl when compared to PPh_3_.

## 1. Introduction

Metal-based drugs are an important tool in the development of new therapeutic drugs. Auranofin, the successful gold(I)-based drug, exhibits both high potency antiarthritic and antitumour properties [1,2]. Auranofin analogues have since been investigated for their interesting coordination to both a phosphine and a thioglucoside. In many cases, *N*-heterocyclic carbenes (NHCs) have been utilized as an alternative to the phosphine ligand [3,4,5]. NHCs have proved to be suitable ligands for stabilizing the highly active gold(I) species, due to their good electron donating ability and their highly stable carbene from π-backbonding [6,7]. As a result, several metal NHC complexes have reported strong anticancer activity [8,9,10].

**Figure 1:**
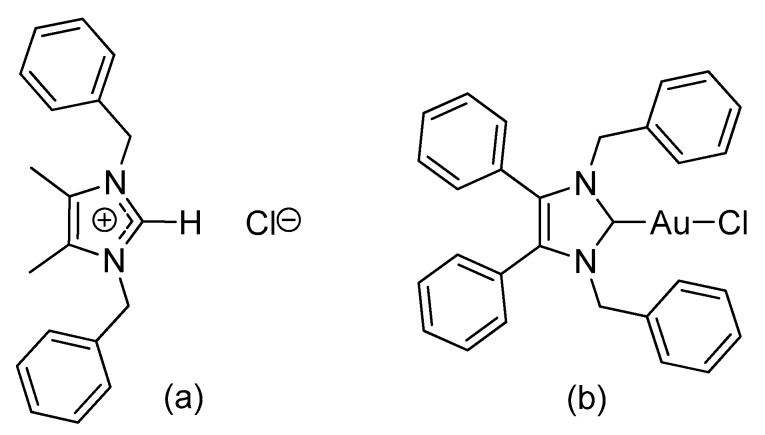
Structure of lepidiline A (1,3-dibenzyl-4,5-dimethylimidazolum chloride) (**a**) and NHC*-Au(I)-Cl (**1**) (**b**).

Lepidiline A (Figure 1), a naturally occurring imidazolium compound extracted from the root of *Lepidium meyenii*, has presented many biological properties, including cytotoxicity [11]. Lepidiline A exhibits activity against the human ovarian cancer cell line FDIGROV, with an ED_50_ of 7.39 μg/mL [11]. Furthermore, this biologically active imidazolium compound acts as a promising structural motif for NHC derivatives [12], and more effective applications of lepidiline A may lie in the development of metal-based complexes with lepidiline A as the coordinating ligand.

Gold(I) complexes are an important class of anticancer drugs, due to their unique mechanism of action. It has been shown that gold(I) complexes can elicit tumour cell death through targeting members of the intracellular redox-homeostasis system, such as the mitochondria associated thioredoxin reductase (TrxR), whose inhibition leads to reactive oxygen species formation [13,14,15]. A selenocysteine–cysteine bridge at the C-terminal of the TrxR enzyme acts as the target for gold(I) [9,16]. Gold(I) has a high affinity for thiols, due to their soft nature, resulting in strong Au–S bonds. However, gold(I) also binds strongly to blood thiols such as serum albumin or glutathione, reducing the amount of drug arriving at cancer cells [17]. Therefore, there is a desire to design a gold(I)-NHC complex that has a suitably strong Au–S bond incorporated to lessen the chance of blood thiol conjugation.

The effectiveness of these gold(I)-NHC complexes are still restricted by cell selectivity. Introducing targeting biomolecules to the complex could ensure the drug is delivered directly to the cancer cells, thus minimizing the death of normal cells and increasing the drug’s efficacy [18]. Modifying the coordinating ligand of the NHC-gold(I) complex to include a carboxylic acid would allow increased functionality, such as esters or amides.

Herein we present a structural assessment of NHC-Au(I) complexes, based on (1,3-dibenzyl-4,5-diphenylimidazol-2-ylidene)gold(I) chloride (NHC*-AuCl), (**1**) Figure 1. The synthesis, characterisation, and biological testing of ten new NHC*-gold(I) complexes is reported. The effect of altering the coordinating ligands of the NHC*-gold(I) on the cytotoxicity is investigated via MTT-based proliferation assays. The cytotoxicity studies of these novel compounds have been conducted in vitro against three different tumour cell lines: MCF-7^topo^ (multidrug-resistant breast cancer), HCT-116^wt^, and the p53 knockout mutant HCT-116^−/−^ (colon cancer). These cytotoxicity studies, compared to that of **1**, can provide information on the ideal structures of future gold(I) chemotherapeutic complexes. Additionally, a computational study of **1** can highlight the advantages of employing an NHC ligand, as opposed to a phosphine.

## 2. Results and Discussion

### 2.1. Synthesis and Characterisation

**Scheme 1:**
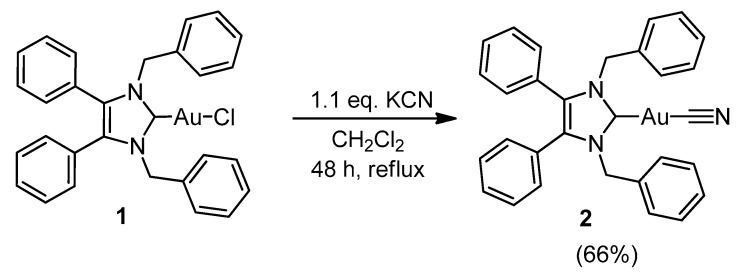
General reaction scheme for the synthesis of NHC*-Au-CN (**2**).

**Scheme 2:**
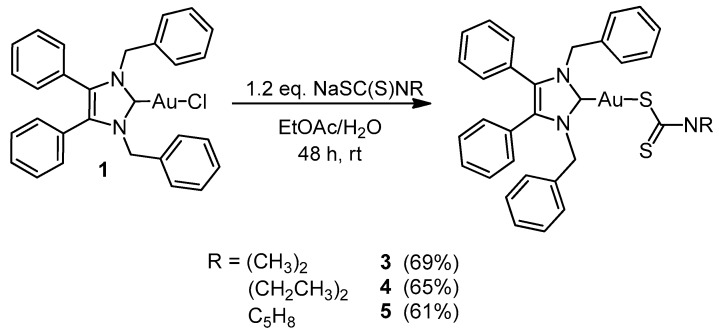
General reaction scheme for the synthesis of NHC*-Au(I) dithiocarbamate complexes **3**–**5**.

**Scheme 3:**
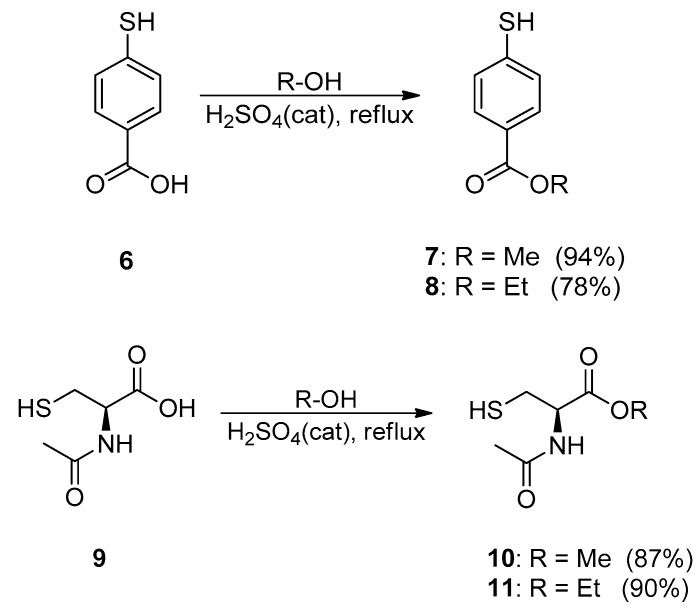
General reaction scheme for the synthesis of esters **7**, **8**, **10**, and **11**.

**Scheme 4:**
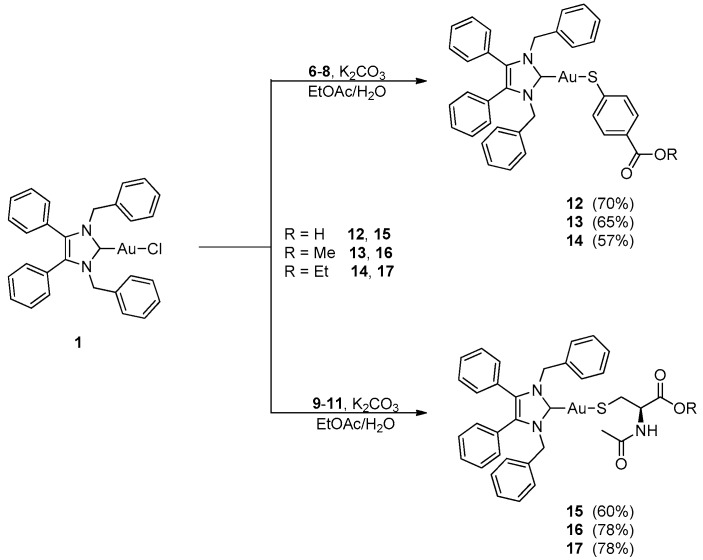
General reaction scheme for the synthesis of NHC*-Au-S-linker (**12**–**17**).

The synthetic route for the ten NHC*-gold(I) complexes described in this paper are shown in Scheme 1, Scheme 2, Scheme 3 and Scheme 4. NHC*-Au(I)-Cl (**1**) was synthesised according to a procedure previously published [3]. The preparation of **1**, *p*-mercaptobenzoate derivatives **7** and **8**, and *N*-acetyl-l-cysteine (NAC) derivatives **10** and **11** (Scheme 3), were confirmed with ^1^H and ^13^C-NMR spectra. Novel complexes **2**–**5** and **12**–**17** were characterised with elemental analysis, high resolution mass spectrometry, IR spectroscopy, and melting point. See Supplementary Material for ^1^H and ^13^C spectra of complexes **2**–**5** and **12**–**17**.

Complex **2** was formed in a 66% yield from the anion exchange of chloride to cyanide (Scheme 1). The precursor **1** was reacted with potassium cyanide in dry dichloromethane at reflux for 48 h to produce complex **2**. The reaction does not form the desired product when conducted in a biphasic solvent system with ethyl acetate and water. Upon reaction in the presence of water, the carbene is protonated to form the corresponding imidazolium dicyanoaurate(I), confirmed by a signal at δ = 8.66 ppm, representing the protonated carbene.

The ^1^H-NMR spectrum of **2** shows a slight shift of the CH_2_ protons of the benzyl groups, from δ = 5.44 ppm to 5.37 ppm, when compared to the ^1^H-NMR of **1**. The quaternary carbon of the cyanide ligand appears in the ^13^C-NMR spectra at δ = 152.6 ppm. An absorption band at 2144 cm^−1^ in the IR spectra of **2** represents the C≡N stretch.

Complexes **3**–**5** were prepared by reacting complex **1** with the corresponding sodium carbamate salt (Scheme 2). This was performed under biphasic conditions by stirring at room temperature in ethyl acetate and water for 48 h, with relatively good yields of 61–69%. Complexes **3**–**5** were also synthesised in dichloromethane at reflux for 24 h, this, however, gave lower yields.

Similar to **2**, the CH_2_ signal in the ^1^H-NMR of complexes **3**–**5** is shifted to δ = 5.57–5.55 ppm upon coordination to the dithiocarbamates. The addition of a new ^1^H-NMR singlet at δ = 3.51 ppm corresponding to the two methyl groups of the dimethyldithiocarbamate moiety (**3**) confirms its coordination to the NHC*-Au(I). Similarly, the CH_2_ and CH_3_ peaks of the diethyldithiocarbamate complex **4** appear at δ = 3.96 ppm and 1.31 ppm, respectively. The pyrrolidine CH_2_ peaks of **5** appear at δ = 3.85 and 1.97 ppm, with a triplet and pentet distinguishing these two peaks. 

**Figure 2:**
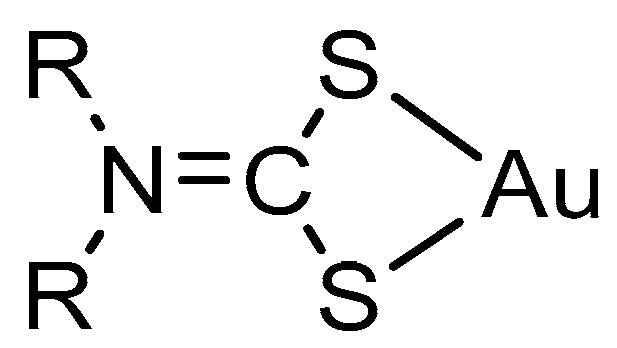
The dominant resonance form of a dithiocarbamate complex.

Previous metal-dialkyldithiocarbamate complexes reported the IR stretch of the carbon sulphur bond from 820–1050 cm^−1^ [19,20]. The IR spectra of **3**, **4** and **5** show a medium band at 971, 910, and 949 cm^−1^, respectively, corresponding to the C=S stretch. A nickel(II) dimethyldithiocarbamate complex exhibited a carbon–sulphur bond stretch at 975 cm^−1^ [19,21], which correlates well with the dimethyldithiocarbamate complex **3**. IR spectra of **3**, **4**, and **5** show bands at 1447, 1411, and 1406 cm^−1^, respectively, which correspond to the carbon–nitrogen stretching of the carbamate. Interestingly, these IR values account for an intermediate bond in the 1450–1550 cm^−1^ range [20]. This indicates a resonance structure is present where the carbon–nitrogen bond exhibits more double bond character than the carbon–sulphur bonds. Furthermore, the presence of only one band for the C=S bond implies the molecule is symmetrical, and therefore, in the resonant structure shown in Figure 2 [19].

The synthetic route to ester formation is highlighted below in Scheme 3. The esters **7**, **8**, **10**, and **11** were made with Fischer esterification, by refluxing 4-mercaptobenzoic acid (**6**) and *N*-acetyl-l-cysteine (NAC) (**9**) (both commercially available) in methanol and ethanol with a catalytic amount of sulphuric acid, to make their corresponding methyl and ethyl esters. Compounds **6**–**11** were conjugated with complex **1**, under basic conditions, to obtain complexes **12**–**17**, in relatively good yields of 57–78% (Scheme 4).

Compounds **6** and **9** were initially conjugated to **1** to make the corresponding NHC*-Au-S-linker molecules **12** and **15**. Esterification of the acid ends of **12** and **15** was unsucessful. Attempts were made to synthesise complexes **13**, **14**, **16**, **17** by reacting **12** and **15** with methanol or ethanol; however, this also proved to be unsucessful. Due to the lack of success via the linear synthesis, we moved to convergent synthesis, which was successful.

The most diagnostic feature in the ^1^H-NMR spectra of complexes **12**–**17** is the disappearance of the SH signal of the thiols once coordinated to the gold. This appears in the δ = 3.64–2.48 ppm range for the *p*-mercaptobenzoate compounds (**12**–**14**) ,and δ = 1.33–1.31 ppm range for the NAC compounds (**15**–**17**). In the NAC series, the acetyl protons on the nitrogen atom of compounds **10** and **11** are observed at δ = 2.07 and 2.09 ppm, respectively. However, once linked to the NHC*-Au(I) centre, there is an observed upfield chemical shift of the acetyl protons to δ = 1.95 and 1.94 ppm in compounds **16** and **17**, respectively. For complexes **13** and **16** there is a slight upfield shift of the CH_3_ singlet of the methyl compounds upon coordination to the gold; however, in the ethyl compounds, a downfield shift is noted.

### 2.2. Structural Discussion

**Figure 3:**
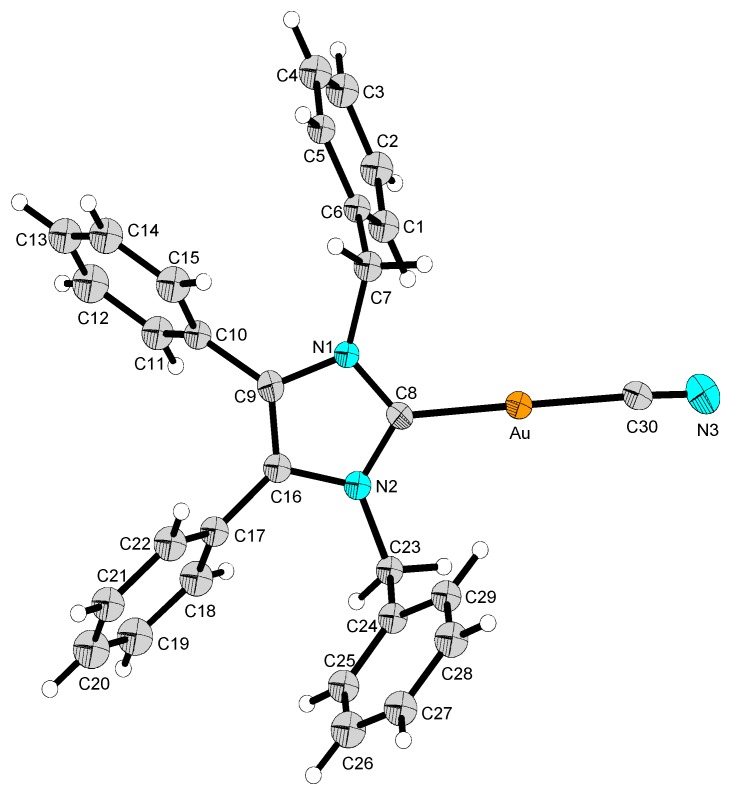
X-ray diffraction structure of NHC*-Au-CN (**2**); thermal ellipsoids are drawn on the 50% probability level.

**Figure 4:**
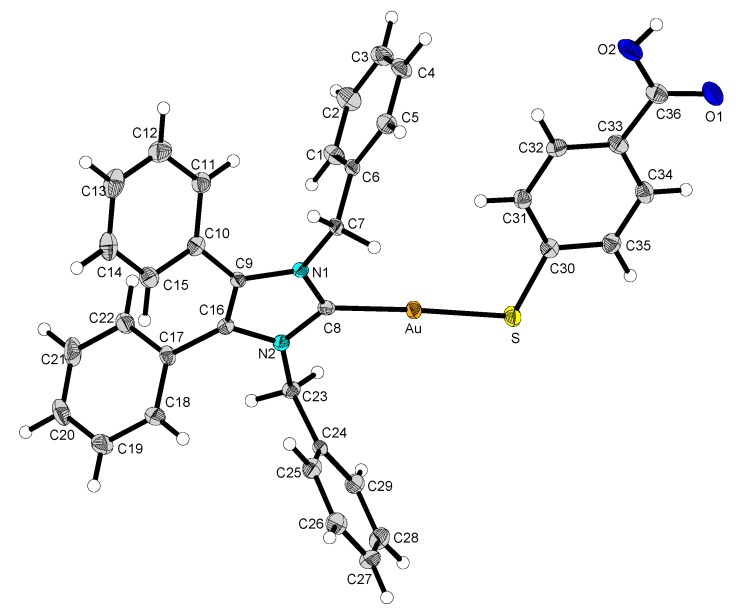
X-ray diffraction structure of **12**; thermal ellipsoids are drawn on the 50% probability level.

**Figure 5:**
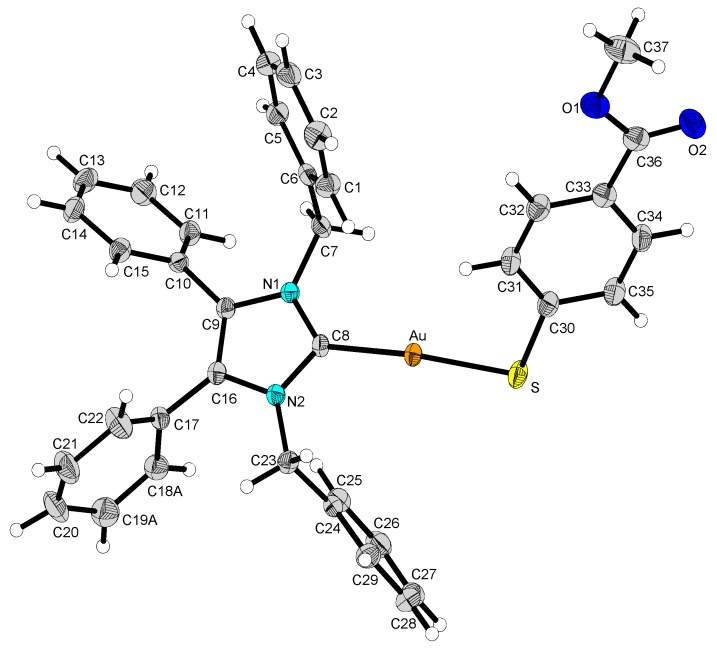
X-ray diffraction structure of **13**; thermal ellipsoids are drawn on the 50% level, disorder neglected.

**Figure 6:**
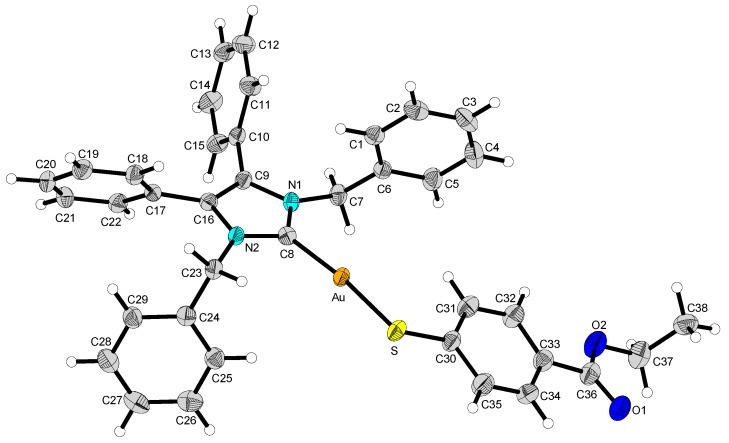
X-ray diffraction structure of **14**; thermal ellipsoids are drawn on the 50% probability level.

X-ray crystallography data was obtained for four of the complexes synthesised. The crystal of complex **2** was developed from the slow diffusion of pentane into a saturated dichloromethane solution at −18 °C. Complex **2** crystallised in the monoclinic space group P2_1_/m (#11) (Figure 3). The crystals of **12** and **13** were formed in a saturated solution of ethyl acetate with the slow infusion of pentane (Figure 4 and Figure 5). Both crystallised in the triclinic space group P$\bar{1}$ (#2), in the absence of any solvent molecules. Crystal **14** was formed in a saturated solution of dichloromethane with slow infusion of diethyl ether (Figure 6). Complex **14** crystallized in the monoclinic space group C2/c (#15), also in the absence of any solvent molecules. The X-ray crystal data and structure refinement of complexes **2**, **12**, **13**, and **14** are found in Table 1, with the selected bond lengths and bond angles compiled in Table 2 and Table 3.

The Au–C(8) bond lengths of 2.031(8) Å for **2**, 2.012(3) Å for **12**, 2.008(2) Å for **13** and 2.008(3) Å for **14** suggest that the gold is strongly bound to the carbene in all four complexes. Additionally, the Au–S bond distance of 2.2856(7) Å in **12**, 2.2851(6) Å in **13** and 2.3012(8) Å in **14** is within the range of reported Au–S bond lengths [22,23]. The C(30)–N(3) bond of **2** of 1.113 Å is indicative of the triple bond of the cyanide ligand [24]. The X-ray structures of all four compounds show an almost linear bond angle of 179.6(4)° for **2**, 177.48(8)° for **12**, 175.20(6)° for **13**, and 173.45(9)° for **14** for the C(8)–Au–C(30) and C(8)–Au–S angles, respectively. Bond angles of 108.40(10)° for **12**, 109.44(8)° for **13**, and 108.83(12)° for **14** are observed for the Au–S–C(30) angle. These values are in good agreement with similar compounds reported earlier by the Tacke group [25,26].

### 2.3. Biological Evaluation

The in vitro anticancer activity of **2**–**5** and **12**–**17** was tested via MTT-based proliferation assays against the human colon carcinoma cell line HCT-116^wt^, the p53 knockout mutant HCT-116^−/−^, and the multidrug-resistant (*mdr*) human breast cancer cell line MCF-7^topo^ (Table 4). Bar **2** and **13**, all complexes reached low single-digit micromolar IC_50_ values against the tested cell lines after 72 h of treatment. These two complexes exhibit only moderate toxicities with IC_50_ values up to 20 µM. While the IC_50_ values of the dithiocarbamate complexes **3**–**5** and the *p*-mercaptobenzoate complexes **12**–**14** vary depending on the nitrogen substitution, and the respective esterification, the complexes carrying NAC, **15**–**17**, show single-digit IC_50_ values in the low micromolar range for all tested cell lines, with almost similar cytotoxic activities throughout. Esterification of NAC with methanol or ethanol slightly increased the antitumor activity against all three cell lines. Amongst the three types of thiolated complexes, the dithiocarbamate complexes **3**–**5** showed the highest activity against the *mdr* MCF-7^topo^ breast cancer cells, with complex **3** being the most active complex in total, with IC_50_ values of 1.5 ± 0.1 µM against the HCT-116^wt^ or 0.28 ± 0.03 µM against the MCF-7^topo^ cells. To test the complexes for their dependency on fully functional p53, one activator of the apoptotic cascade, the complexes were tested for their toxicity against a HCT-116 p53 knockout mutant. Surprisingly, only a few of the tested complexes showed similar or higher IC_50_ values against the knockout mutant than against the wildtype cells. Complexes **4**, **5**, **12**, and **13** exert a higher toxicity against the HCT-116^−/−^ than against the wildtype HCT-116^wt^. Overall, the herein presented complexes exhibit high to moderate antitumoral activity against colon carcinoma cells and a *mdr* breast cancer cell line. Dithiocarbamate complex **3** shows the overall highest activity in all tested cell lines. 

### 2.4. Computational Results

**Figure 7:**
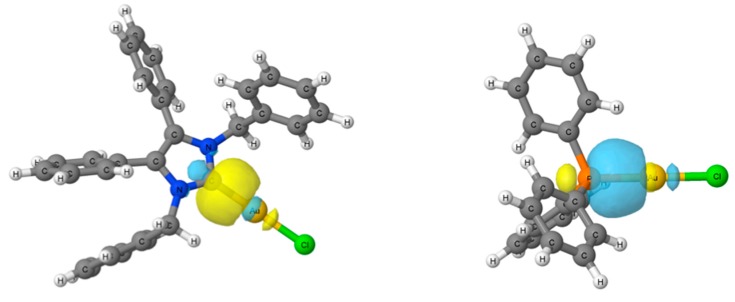
Molecular orbital corresponding to the σ-C–Au and σ-P–Au bonds of NHC*-AuCl and Ph_3_P-AuCl respectively.

The enthalpy of formation has been obtained at the MN15/6-311++G(2df,p)/LANL2TZ(f) level for both NHC*-AuCl and Ph_3_P-AuCl compounds (Figure 7). The results show that NHC*-AuCl presents a more negative enthalpy (−315.0 kJ/mol) than Ph_3_P-AuCl (−274.3 kJ/mol), which indicates that the formation of NHC*-AuCl is more favourable. Natural bond orbital (NBO) analysis shows that the AuCl unit in NHC*-AuCl is slightly less negative (−0.32e^−^) than in Ph_3_P-AuCl (−0.35e^−^), and displays slightly shorter Au–Cl distances in NHC*-AuCl (2.291 Å) than in Ph_3_P-AuCl (2.299 Å). This is indicative of a stronger bond between the gold and the carbene due to the higher σ-donating effect of the nucleophilic NHC* ligand.

Also, for NHC*-AuCl, two backbonding donations from the gold into the π* C–N antibonding orbitals are observed, E(2) = 15.9 and 16.1 kJ/mol; while in Ph_3_P-AuCl, three backbonding donations are observed from the Au atom into the π* P–C antibonding orbitals with E(2) = 16.2, 16.0, and 14.9 kJ/mol. The additional backbonding in the Ph_3_P-AuCl molecule reduces its bond strength, resulting in a weaker donating ligand. Conclusively, these results give credence to NHCs being the more favourable ligand than phosphines.

## 3. Materials and Methods

### 3.1. General Conditions

All chemicals were purchased and used as received, unless otherwise stated. Solvents were dried according to the standard procedures, when necessary. ^1^H and ^13^C spectra were recorded on either a 300 or 400 MHz Varian spectrometer at room temperature (rt). Both chloroform (CDCl_3_) and dimethyl sulfoxide (DMSO) were used as deuterated solvents. The residual solvent peak or tetramethylsilane (TMS) were used as the internal standard. All chemical shifts are reported as δ values in parts per million (ppm). Infrared spectra were recorded on a Bruker ALPHA PLATINUM ATR spectrometer (Millerica, MA, USA). High resolution accurate mass data were obtained on a Waters/Micromass LCT TOF spectrometer (Milford, MA, USA). under electrospray ionisation technique. Melting points were measured on a Stuart™ (Stone, UK). melting point apparatus SMP10. Elemental analysis was conducted on an Exeter Analytical CE-440 elemental analyser (Coventry, UK). X-ray crystallography data was collected on a Rigaku Oxford Diffraction (Chalgrove, UK) SuperNova A diffractometer. Absorbance measurements were done with a TECAN (Männedorf, Switzerland) Infinite F200 plate reader.

### 3.2. Synthesis

#### 3.2.1. (1,3-Dibenzyl-4,5-diphenylimidazol-2-ylidene)gold(I) Chloride (**1**)

Prepared according to literature method [3]. ^1^H-NMR (300 MHz, CDCl_3_, δ ppm): 7.30 (t, *J* = 7.4 Hz, 2H, CH_benzyl_), 7.25–7.16 (m, 10H, CH_benzyl_ + CH_phenyl_), 7.06–6.92 (m, 8H, CH_phenyl_), 5.44 (s, 4H, CH_2_). ^13^C-NMR (101 MHz, CDCl_3_, δ ppm): 171.6 (NCN), 135.8, 132.2, 130.9, 129.5, 128.8, 128.7, 128.2, 127.6, 127.4 (CH_imidazol_ + CH_phenyl_ + CH_benzyl_), 53.2 (CH_2_).

#### 3.2.2. (1,3-Dibenzyl-4,5-diphenyl-2-ylidene)gold(I) Cyanide (**2**)

(1,3-Dibenzyl-4,5-diphenylimidazol-2-ylidene)gold(I) chloride (65 mg, 0.10 mmol) and potassium cyanide (7.5 mg, 0.12 mmol) were stirred in dichloromethane (15 mL) under reflux for 48 h. The reaction was washed with deionised water (2 × 10 mL). The organic solution was extracted and dried over anhydrous MgSO_4_. This was filtered, and the excess solvent reduced under pressure to 3 mL. Pentane (40 mL) was added to precipitate a white solid. The product was filtered, washed with pentane (15 mL), and dried in vacuo. Yield: 40.8 mg, 66%. ^1^H-NMR (400 MHz, CDCl_3_, δ ppm): 7.32 (t, *J* = 7.4 Hz, 2H, CH_benzyl_), 7.25–7.20 (m, 10H, CH_benzyl_ + CH_phenyl_), 6.99 (t, *J* = 5.9 Hz, 8H, CH_phenyl_), 5.37 (s, 4H, CH_2_). ^13^C-NMR (101 MHz, CDCl_3_, δ ppm): 182.9 (NCN), 152.6 (CN), 135.7, 132.6, 130.9, 129.7, 128.9, 128.8, 128.4, 127.6, 127.2 (C_imidazol_ + C_phenyl_ + C_benzyl_), 53.0 (CH_2_). IR (ATR): 3058 (w), 3030 (w), 2143 (w), 1594 (w), 1488 (m), 1447 (m), 1348 (m), 1026 (m), 758 (m), 696 (s). MS (ESI^+^) *m/z*: 624.2 [M + H]^+^. Melting point range: 264–268 °C. Anal. calcd for C_30_H_24_AuN_3_ (623.51): C, 57.79; H, 3.88; N, 6.74. Found: C, 61.08; H, 4.04; N, 6.90. Although these elemental results are outside the acceptable range to establish purity, they demonstrate the best results yet obtained.

#### 3.2.3. General Procedure for NHC-Au(I) Complexes **3**–**5**

(1,3-Dibenzyl-4,5-diphenylimidazol-2-ylidene)gold(I) chloride (65 mg, 0.10 mmol) and the corresponding sodium carbamate salt (0.12 mmol) were stirred in a biphasic solution of ethyl acetate (7 mL) and deionised water (6 mL) at rt for 48 h. The reaction mixture was washed with deionised water (2 × 10 mL) and an aqueous saturated solution of NaCl (10 mL). The combined organic phase was dried over anhydrous MgSO_4_, filtered, and reduced to approximately 3 mL under reduced pressure. Pentane (40 mL) was added to precipitate a solid. The product was filtered, washed with pentane (15 mL), and dried in vacuo.

##### (1,3-Dibenzyl-4,5-diphenylimidazol-2-ylidene)gold(I) Dimethyldithiocarbamate (**3**)

A white solid was formed. Yield: 50.4 mg, 69%. ^1^H-NMR (300 MHz, CDCl_3_, δ ppm): 7.32–7.27 (m, 2H, CH), 7.24–7.16 (m, 10H, CH), 7.10–7.04 (m, 4H, CH), 6.95 (d, *J* = 7.1 Hz, 4H, CH), 5.55 (s, 4H, CH_2_), 3.51 (s, 6H, CH_3_). ^13^C-NMR (101 MHz, CDCl_3_, δ ppm): 136.9, 132.4, 131.2, 131.1, 129.6, 129.1, 128.9, 128.3, 127.1 (C_imidazol_ + C_phenyl_ + C_benzyl_), 53.0 (CH_2_), 45.4 (CH_3_). MS (ESI^+^) *m/z*: 718.2 [M + H]^+^. IR (ATR): 3025 (w), 2910 (w), 1603 (w), 1496 (m), 1447 (m), 1248 (m), 1140 (m), 971 (m), 726 (m), 695 (s). Melting point range: 186–187 °C. Anal. calcd for C_32_H_30_N_3_S_2_Au (717.71): C, 53.55; H, 4.21; N, 5.85; S, 8.94. Found: C, 53.50; H, 4.17; N, 5.77; S, 8.64.

##### (1,3-Dibenzyl-4,5-diphenylimidazol-2-ylidene)gold(I) Diethyldithiocarbamate (**4**)

A white solid was formed. Yield: 49.5 mg, 65%. ^1^H-NMR (300 MHz, CDCl_3_, δ ppm): 7.27 (t, *J* = 7.3 Hz, 2H, CH), 7.25–7.14 (m, 15H, CH), 7.08–7.04 (m, 3H, CH), 6.93 (d, *J* = 7.2 Hz, 3H, CH), 5.57 (s, 4H, CH_2_-_Bz_), 3.96 (q, *J* = 7.1 Hz, 4H, CH_2-ethyl_), 1.31 (t, *J* = 7.0 Hz, 6H, CH_3_). ^13^C-NMR (101 MHz, CDCl_3_, δ ppm): 205.8 (SCS), 180.1 (NCN), 136.3, 132.1, 130.9, 129.2, 128.6, 128.5, 127.9, 127.8 (CH_imidazol_ + CH_phenyl_ + CH_benzyl_), 53.1 (CH_2-benzyl_), 49.3 (CH_2-ethyl_), 12.4 (CH_3_). MS (ESI^+^) *m/z*: 746.2 [M + H]^+^. IR (ATR): 3025 (w), 2925 (w), 1603 (w), 1495 (w), 1411 (m), 1260 (m), 1133 (m), 981 (m), 910 (m), 733 (s), 694 (s). Melting point range: 187–188 °C. Anal. calcd for C_34_H_34_N_3_S_2_Au (745.75): C, 54.76; H, 4.60; N, 5.63; S, 8.60. Found: C, 54.58; H, 4.52; N, 5.53; S, 8.72.

##### (1,3-Dibenzyl-4,5-diphenylimidazol-2-ylidene)gold(I) Pyrrolidinedithiocarbamate (**5**)

A white solid was formed. Yield: 46.6 mg, 61%. ^1^H-NMR (300 MHz, CDCl_3_, δ ppm): 7.29 (d, *J* = 7.5 Hz, 1H, CH), 7.24–7.14 (m, 10H, CH), 7.12–7.03 (m, 4H, CH), 6.95 (d, *J* = 7.0 Hz, 4H, CH), 5.56 (s, 4H, CH_2-Bz_), 3.85 (t, 4H, CH_2_), 1.97 (p, 4H, CH_2_). ^13^C-NMR (101 MHz, CDCl_3_, δ ppm): 202.8 (SCS), 180.2 (NCN), 136.3, 132.1, 130.9, 129.3, 128.6, 128.5, 128.0, 127.9, 127.8 (CH_imidazol_ + CH_phenyl_ + CH_benzyl_), 54.43 (CH_2_), 53.08 (CH_2-benzyl_), 26.30 (CH_2_). MS (ESI^+^) *m/z*: 744.2 [M + H]^+^. IR (ATR): 3027 (w), 2961 (w), 1602 (w), 1494 (w), 1406 (m), 1165 (m), 949 (m), 733 (m), 695 (s). Melting point range: 188–189 °C. Anal. calcd for C_34_H_32_N_3_S_2_Au (743.73): C, 54.91; H, 4.34; N, 5.65; S, 8.63. Found: C, 54.48; H, 4.26; N, 5.52; S, 8.85.

#### 3.2.4. General Procedure for **7**–**8**, **10**–**11**

Esters **7**–**8** and **10**–**11** were prepared according to modified literature methods [27,28]. The carboxylic acid (**6** or **9**) was dissolved in either methanol (30 mL) or ethanol (30 mL), with 2 drops of concentrated sulfuric acid added to the solution before refluxing at 90 °C for 24 h. The reaction progress was monitored by TLC (cyclohexane-ethyl acetate; 1:1). The reaction was concentrated under reduced pressure to yield a white solid.

##### Methyl-*p*-mercaptobenzoate (**7**)

The residue was purified with column chromatography (cyclohexane-ethyl acetate; 3:1) to produce a white solid. Yield: 319 mg, 95%. The NMR data were in agreement with those reported in literature [27,28]. ^1^H-NMR (400 MHz, CDCl_3_, δ ppm): 7.88 (d, *J* = 8.6 Hz, 2H, CH), 7.27 (d, *J* = 8.6 Hz, 2H, CH), 3.89 (s, 3H, CH3), 3.60 (s, 1H, SH). ^13^C-NMR (101 MHz, CDCl_3_, δ ppm): 166.9 (C=O), 138.3 (CH), 130.2 (CH), 128.1 (CH), 127.1 (CH), 52.0 (CH_3_).

##### Ethyl-*p*-mercaptobenzoate (**8**)

The residue was purified with column chromatography (cyclohexane-ethyl acetate; 3:1) to produce a white solid. Yield: 319 mg, 95%. The NMR data were in agreement with those reported in literature [29]. ^1^H-NMR (400 MHz, DMSO-*d_6_*, δ ppm): 7.77 (d, *J* = 8.5 Hz, 2H, CH), 7.40 (d, *J* = 8.5 Hz, 2H, CH), 4.26 (q, *J* = 7.1 Hz, 2H, CH_2_), 2.48 (s, 2H, SH), 1.28 (t, *J* = 7.1 Hz, 3H, CH_3_). ^13^C-NMR (101 MHz, DMSO-*d_6_*, δ ppm): 165.4 (C=O), 141.7 (CH), 130.5 (CH), 129.1 (CH), 126.5 (CH), 61.2 (CH_2_), 14.5 (CH_3_).

##### *N*-Acetyl-l-cysteine Methyl Ester (**10**)

The crude product was used without further purification as a white solid. Yield: 618 mg, 88%. The NMR data were in agreement with those reported in literature [30]. ^1^H-NMR (400 MHz, CDCl_3_, δ ppm): 4.89 (dt, *J* = 7.8, 4.1 Hz, 1H, CH), 3.79 (s, 3H, OCH_3_), 3.01 (ddd, *J* = 9.0, 4.1, 2.7 Hz, 2H, CH_2_), 2.07 (s, 3H, CH_3_), 1.33 (t, *J* = 9.0 Hz, 1H, SH). ^13^C-NMR (101 MHz, CDCl_3_, δ ppm): 170.5 (C=O), 170.0 (C=O), 53.5 (CH), 52.8 (OCH_3_), 26.8 (CH_2_), 23.1 (CH_3_).

##### *N*-acetyl-l-cysteine Ethyl Ester (**11**)

The crude product used without further purification as a white solid. Yield: 650 mg, 85%. The NMR data were in agreement with those reported in literature [31]. ^1^H-NMR (400 MHz, DMSO-*d_6_*, δ ppm): 4.91–4.82 (m, 2H, CH_2_), 4.32–4.19 (m, 2H, OCH_2_), 3.15–2.94 (m, 3H, CH_3_), 2.09 (s, 3H, CH_3_), 1.31 (t, *J* = 7.1 Hz, 1H, SH). ^13^C-NMR (101 MHz, DMSO-*d_6_*, δ ppm): 172.1 (C=O), 169.8 (C=O), 61.9 (OCH_2_), 54.7 (CH), 51.7 (CH_3_), 26.0 (CH_2_), 22.8 (CH_3_).

#### 3.2.5. (1,3-Dibenzyl-4,5-diphenylimidazol-2-ylidene)gold(I) *p*-Mercaptobenzoic Acid (**12**)

(1,3-Dibenzyl-4,5-diphenylimidazol-2-ylidene)gold(I) chloride (63 mg, 0.10 mmol) and *p*-mercaptobenzoic acid (31 mg, 0.20 mmol) were dissolved in ethyl acetate (5 mL), and K_2_CO_3_ (27 mg, 0.20 mmol) was dissolved in water (5 mL). Both solutions were mixed and stirred vigorously at rt for 24 h. The two phases were separated, and the aqueous phase was re-extracted twice with ethyl acetate (10 mL). The combined organic phase was washed with 8% HCl (2 × 10 mL), before drying over MgSO_4_ and filtered. The filtrate was concentrated to approximately 3 mL before the addition of pentane (40 mL). The solution was cooled down to −26 °C to allow the product to precipitate out of the solution before filtering and drying in vacuo. An off-white product was isolated. Yield: 55 mg, 70%. ^1^H-NMR (400 MHz, CDCl_3_, δ ppm): 7.63 (d, *J* = 8.4 Hz, 2H, Hb), 7.47 (d, *J* = 8.4 Hz, 2H, Ha), 7.34–7.18 (m, 12H, CH), 7.09–6.96 (m, 8H, CH), 5.45 (s, 4H, CH_2-benzyl_). ^13^C-NMR (101 MHz, CDCl_3_, δ ppm): 182.3 (NCN), 171.7 (C=O), 153.7, 135.8, 132.0, 131.8, 130.6, 129.4, 129.3, 128.6, 128.6, 128.1, 127.3, 127.2, 123.1 (CH_imidazol_ + CH_phenyl_ + CH_benzyl_), 52.6 (CH_2_). MS (QMS-MS/MS) *m/z*: 773.15 [M + Na]^+^. IR (ATR): 3056 (w), 1668 (w), 1580 (w), 1487 (m), 1446 (w), 1025 (m), 764 (m), 729 (s), 694 (s), 628(w), 518 (w). Melting point range: 177–179 °C. Anal. calcd for C_36_H_29_N_2_O_2_SAu (750.70): C, 57.59; H, 3.90; N, 3.73; S, 4.27; Found: C, 57.33; H, 3.72; N, 3.60; S, 4.59.

#### 3.2.6. (1,3-Dibenzyl-4,5-diphenylimidazol-2-ylidene)gold(I)-methyl-*p*-mercaptobenzoate (**13**)

(1,3-Dibenzyl-4,5-diphenylimidazol-2-ylidene)gold(I) chloride (253 mg, 0.40 mmol) and methyl-*p*-mercaptobenzoate (218 mg, 1.60 mmol) were dissolved in ethyl acetate (20 mL), and potassium carbonate (222 mg, 1.60 mmol) was dissolved in water (20 mL). Both solutions were mixed and stirred vigorously at rt for 24 h. The two phases were separated, and the aqueous phase was washed with ethyl acetate (2 × 20 mL). The organic phases were combined and washed with 8% HCl (20 mL), an aqueous saturated solution of NaHCO_3_ (20 mL) and an aqueous saturated solution of NaCl (20 mL). The organic phase was dried over MgSO_4_, filtered, and concentrated to approximately 5 mL before the addition of pentane (40 mL). The solution was cooled down to −20 °C to allow the product to precipitate out of the solution before filtering and drying in vacuo. An off-white product was isolated. Yield: 202 mg, 65%. ^1^H-NMR (400 MHz, CDCl_3_, δ ppm): 7.62–7.57 (m, 2H, Hb), 7.51–7.46 (m, 2H, Ha), 7.34–7.28 (m, 2H, CH), 7.27–7.21 (m, 10H, CH), 7.08–7.04 (m, 4H, CH), 7.03–6.98 (m, 4H, CH), 5.46 (s, 4H, CH_2_), 3.84 (s, 3H, OCH_3_). ^13^C-NMR (101 MHz, CDCl_3_, δ ppm): 182.3 (NCN), 167.9 (C=O), 135.8, 132.0, 131.8, 130.6, 129.3, 128.8, 128.6, 128.6, 128.1, 127.3, 127.2, 124.1 (CH_imidazol_ + CH_phenyl_ + CH_benzyl_), 52.6 (CH_2_), 51.6 (OCH_3_). MS (QMS-MS/MS) *m/z*: 765.18 [M + H]^+^. IR (ATR): 3057 (w), 1705 (s), 1584 (s), 1432 (m), 1279 (s), 1270 (s), 1172 (w), 1107 (m), 1085 (m), 1021 (w), 760 (s), 696 (s), 526 (w). Melting point range: 149–152 °C. Anal. calcd for C_37_H_31_N_2_O_2_AuS (764.73): C, 58.11; H, 4.09; N, 3.66; S, 4.19. Found: C, 58.28; H, 4.02; N, 3.41; S, 4.20.

#### 3.2.7. (1,3-Dibenzyl-4,5-diphenylimidazol-2-ylidene)gold(I)-ethyl-p-mercaptobenzoate (**14**)

(1,3-Dibenzyl-4,5-diphenylimidazol-2-ylidene)gold(I) chloride (254 mg, 0.40 mmol) and ethyl-*p*-mercaptobenzoate (291 mg, 1.60 mmol) were dissolved in ethyl acetate (20 mL), and potassium carbonate (221 mg, 1.60 mmol) was dissolved in water (20 mL). Both solutions were mixed and stirred vigorously at rt for 24 h. The two phases were separated, and the aqueous phase was re-extracted twice with ethyl acetate (20 mL). The organic phases were combined and washed with 8% HCl (20 mL), an aqueous saturated solution of NaHCO_3_ (20 mL), and an aqueous saturated solution of NaCl (20 mL). The organic phase was dried over MgSO_4_, filtered, and concentrated to approximately 5 mL before the addition of pentane (40 mL). The solution was cooled down to −20 °C to allow the product to precipitate out of the solution before filtering and drying in vacuo. An off-white product was isolated. Yield: 178 mg, 57%. ^1^H-NMR (300 MHz, CDCl_3_, δ ppm): 7.61 (d, *J* = 8.4 Hz, 2H, Hb), 7.48 (d, *J* = 8.4, 2H, Ha), 7.35–7.27 (m, 2H, CH), 7.25–7.17 (m, 10H, CH), 7.09–6.93 (m, 8H, CH), 5.44 (s, 4H, CH_2-benzyl_), 4.30 (q, *J* = 7.1 Hz, 2H, CH_2-ethyl_), 1.35 (t, *J* = 7.1 Hz, 3H, CH_3_). ^13^C-NMR (101 MHz, CDCl_3_), δ ppm): 166.9 (C=O), 135.6, 132.0, 131.8, 130.6, 129.3, 128.8, 128.5, 128.0, 127.4, 127.2 (CH_imidazol_ + CH_phenyl_ + CH_benzyl_), 60.3 (CH_2-ethyl_), 52.7 (CH_2-benzyl_), 14.3 (CH_3_). MS (QMS-MS/MS) *m/z*: 779.20 [M + H]^+^. IR (ATR): 3056 (w), 1698 (m), 1585 (m), 1445 (m), 1277 (m), 1267 (m), 1105 (m), 1092 (m), 763 (m), 729 (s), 694 (s), 527 (m). Melting point range: 163–166 °C. Anal. calcd for C_38_H_33_N_2_O_2_AuS (778.76): C, 58.60; H, 4.28; N, 3.60; S, 4.11. Found: C, 58.45; H, 4.01; N, 3.74; S, 4.32.

#### 3.2.8. (1,3-Dibenzyl-4,5-diphenylimidazol-2-ylidene)gold(I)-*N*-acetyl-l-cysteine (**15**)

(1,3-Dibenzyl-4,5-diphenylimidazol-2-ylidene)gold(I) chloride (63 mg, 0.10 mmol) and *N*-acetyl-l-cysteine (18 mg, 0.11 mmol) were dissolved in ethyl acetate (5 mL), and potassium carbonate (15 mg, 0.11 mmol) was dissolved in water (5 mL). Both solutions were mixed and stirred vigorously at rt for 24 h. The two phases were separated, and the aqueous phase was washed with ethyl acetate (2 × 10 mL). The combined organic phase was washed with 8% HCl (2 × 10 mL), then dried over MgSO_4_ and filtered. The filtrate was concentrated to approximately 3 mL before the addition of pentane (40 mL). The solution was cooled down to −20 °C to allow the product to precipitate out of the solution before filtering and drying in vacuo. An off-white product was isolated. Yield: 45 mg, 60%. ^1^H-NMR (300 MHz, CDCl_3_, δ ppm): 7.35–7.08 (m, 12H, CH), 7.04–6.88 (m, 8H, CH), 5.44–5.28 (m, 4H, CH_2-benzyl_), 4.51 (q, *J* = 4.1 Hz, 1H, CH), 3.91 (s, 2H, CH_2-NAC_), 1.88 (s, 3H, CH_3_). ^13^C-NMR (101 MHz, CDCl_3_, δ ppm): 178.0 (NCN), 172.1 (C=O), 169.6 (C=O), 136.2, 132.0, 130.6, 129.2, 128.5, 128.4, 127.8, 127.5, 127.2 (CH_imidazol_ + CH_phenyl_ + CH_benzyl_), 56.8 (CH_2-NAC_), 52.5 (CH_2-benzyl_), 23.6 (CH_3_). MS (QMS-MS/MS) *m/z*: 760.59 [M + H]^+^. IR (ATR): 3057 (w), 3030 (w), 1665 (m), 1495 (m), 1447 (w), 1075 (w), 1022 (), 764 (m), 730 (m), 696 (s), 518 (w). Melting point range: 102–105 °C. Anal. calcd for C_34_H_32_N_3_O_3_AuS (759.72): C, 53.75; H, 4.25; N, 5.53; S, 4.22. Found: C, 53.42; H, 4.34; N, 5.13; S, 3.94.

#### 3.2.9. (1,3-Dibenzyl-4,5-diphenylimidazol-2-ylidene)gold(I)-*N*-acetyl-l-cysteine Methyl Ester (**16**)

(1,3-Dibenzyl-4,5-diphenylimidazol-2-ylidene)gold(I) chloride (253 mg, 0.40 mmol) and *N*-acetyl-l-cysteine methyl ester (285 mg, 1.60 mmol) were dissolved in ethyl acetate (20 mL), and potassium carbonate (223 mg, 1.60 mmol) was dissolved in water (20 mL). Both solutions were mixed and stirred vigorously at rt for 24 h. The two phases were separated, and the aqueous phase was washed with ethyl acetate (2 × 20 mL). The organic phases were combined and washed with 8% HCl (20 mL), an aqueous saturated solution of NaHCO_3_ (20 mL), and an aqueous saturated solution of NaCl (20 mL). The organic phase was dried over MgSO_4_, filtered, and concentrated to approximately 5 mL before the addition of pentane (40 mL). The solution was cooled down to −20 °C to allow the product to precipitate out of the solution before filtering and drying in vacuo. An off-white product was isolated. Yield: 242 mg, 78%. ^1^H-NMR (300 MHz, CDCl_3_, δ ppm): 7.34–7.26 (m, 2H, CH), 7.25–7.18 (m, 10H, CH), 7.06–6.93 (m, 8H, CH), 5.43 (s, 4H, CH_2-benzyl_), 4.73 (dt, *J* = 7.5, 4.7 Hz, 1H, CH), 3.65 (s, 3H, OCH_3_), 3.36 (dd, *J* = 13.1, 5.5 Hz, 1H, CH_2_), 3.23 (dd, *J* = 13.1, 4.7 Hz, 1H, CH_2_), 1.95 (s, 3H, CH_3-NAC_). ^13^C-NMR (101 MHz, CDCl_3_, δ ppm): 171.8 (C=O), 169.9 (C=O), 135.9, 131.9, 130.6, 129.2, 128.7, 128.5, 128.0, 127.4, 127.3, 126.5 (CH_imidazol_ + CH_phenyl_ + CH_benzyl_), 55.0 (CH), 52.6 (CH_2-benzyl_), 52.1 (CH_3_), 30.0 (CH_2-NAC_), 23.1 (CH_3-NAC_). MS (QMS-MS/MS) *m/z*: 774.20 [M + H]^+^. IR (ATR): 3058 (w), 1740 (m), 1670 (m), 1496 (m), 1447 (m), 1207 (w), 1022 (w), 764 (m), 733 (m), 698 (s), 518 (w). Melting point range: 77–78 °C. Anal. calcd for C_35_H_34_N_3_O_3_AuS (773.75): C, 54.33; H, 4.44; N, 5.43; S, 4.14. Found: C, 54.61; H, 4.26; N, 5.21; S, 4.44.

#### 3.2.10. (1,3-Dibenzyl-4,5-diphenylimidazol-2-ylidene)gold(I)-*N*-acetyl-l-cysteine Ethyl Ester (**17**)

(1,3-Dibenzyl-4,5-diphenylimidazol-2-ylidene)gold(I) chloride (254 mg, 0.40 mmol) and *N*-acetyl-l-cysteine ethyl ester (306 mg, 1.60 mmol) were dissolved in ethyl acetate (20 mL), and potassium carbonate (221 mg, 1.60 mmol) was dissolved in water (20 mL). Both solutions were mixed and stirred vigorously at rt for 24 h. The two phases were separated, and the aqueous phase was washed with ethyl acetate (2 × 20 mL). The organic phases were combined and washed with 8% HCl (20 mL), an aqueous saturated solution of NaHCO_3_ (20 mL), and an aqueous saturated solution of NaCl (20 mL). The organic phase was dried over MgSO_4_, filtered, and concentrated to approximately 5 mL before the addition of pentane (40 mL). The solution was cooled down to −20 °C to allow the product to precipitate out of the solution before filtering and drying in vacuo. An off-white product was isolated. Yield: 245 mg, 78%. ^1^H-NMR (400 MHz, CDCl_3_, δ ppm): 7.32–7.26 (m, 2H, CH), 7.24–7.18 (m, 10H, CH), 7.05–6.94 (m, 8H, CH), 5.43 (s, 4H, CH_2-benzyl_), 4.69 (dd, *J* = 8.4, 4.0 Hz, 1H, CH), 4.18–4.07 (m, 2H, CH_2_), 3.29–3.19 (m, 2H, CH_2_), 1.94 (s, 3H, CH_3-NAC_), 1.21 (t, *J* = 7.1 Hz, 3H, CH_3-methyl_). ^13^C-NMR (101 MHz, CDCl_3_, δ ppm): 171.3 (NCN), 170.3 (C=O), 169.9 (C=O), 135.9, 131.9, 130.6, 129.2, 128.7, 128.5, 128.5, 127.9, 127.4, 127.4 (CH_imidazol_ + CH_phenyl_ + CH_benzyl_), 61.0 (CH_2-ethyl_), 55.1 (CH), 52.9 (CH_2-benzyl_), 40.9 (CH_2-NAC_), 23.1 (CH_3-NAC_), 14.2 (CH_3-ethyl_). MS (QMS-MS/MS) *m/z*: 788.21 [M + H]^+^. IR (ATR): 3030 (w), 1743 (m), 1660 (m), 1496 (m), 1446 (m), 1202 (m), 1178 (m), 1022 (m), 764 (m), 732 (m), 697 (s), 517 (m). Melting point range: 65–68 °C. Anal. calcd for C_36_H_36_N_3_O_3_AuS (787.78): C, 54.88; H, 4.62; N, 5.33; S, 4.07. Found: C, 54.83; H, 4.61; N, 5.27; S, 4.15.

### 3.3. Structure Determination

X-ray crystallography data was collected on a Rigaku Oxford Diffraction SuperNova A diffractometer. Complex **12** was measured with Mo-K_α_ (0.71073 Å), while complexes **2**, **13**, and **14** were measured with Cu-K_α_ (1.54184 Å). A complete dataset was collected, assuming that the Friedel pairs are not equivalent. An analytical absorption correction based on the shape of the crystal was performed [32]. The structures were solved by direct methods using SHELXS [33] and refined by full matrix least-squares on F2 for all data using SHELXL [33]. Hydrogen atoms were added at calculated positions and refined using a riding model. Their isotropic temperature factors were fixed to 1.2 times (1.5 times for methyl and OH groups) the equivalent isotropic displacement parameters of the parent atom. Anisotropic thermal displacement parameters were used for all non-hydrogen atoms. CCDC 1854008 (**2**), CCDC 1850909 (**12**), CCDC 1850910 (**13**), CCDC 1850908 (**14**) contain the supplementary crystallographic data for this paper, available free of charge from the Cambridge Crystallographic Data Centre via www.ccdc.cam.ac.uk/structures.

### 3.4. MTT-Based Proliferation Assay

The cytotoxic activity of al gold complexes was determined via MTT-based proliferation assays for the colon carcinoma cell line HCT-116^wt^, its p53 knockout mutant HCT-116^−/−^, and the multidrug-resistant MCF-7^topo^ breast cancer cell line. The cells, kept in Dulbecco’s Modified Eagle Medium (1% anti-anti, 10% FBS), were seeded into the wells of a clear 96 well plate (5 × 10^4^ cells/well) and incubated for 24 h at standard cell culture conditions (37 °C, 5% CO_2_, 95% humidity). Appropriate pre-dilutions of freshly made stock solutions (10 mM in DMSO) of **2**–**5**, **12**–**15**, and DMSO as negative control, were added into the wells of the pre-incubated cells. After 72 h, the medium was exchanged for a MTT solution (0.05% in PBS) and the cells were further incubated for 2 h. Thereupon, the MTT solution was again discarded, and the cells and violet formazan were dissolved in an SDS/DMSO solution (1% SDS, 0.6% AcOH). After another incubation time of 1 h at 37 °C, the absorbance of formazan at 570 nm, and the background at 630 nm, were measured. Means and SDs are calculated from four independent measurements.

### 3.5. Computational Details

All compounds have been optimized at the MN15 [34] computational level with the 6–311++G(2df,p) basis set [35] applied to the lighter elements inclusive chlorine. The LANL2TZ(f) basis set [36] is used throughout for the gold atoms. Frequency calculations have been performed at the same level in order to confirm that the structures obtained correspond to energetic minima. The effect of water solvation was then accounted for using the SMD approach implemented in the Gaussian16 [37] package including dispersing, repulsing, and cavitation energy terms of the solvent in the optimisation. Orbitals have been calculates using NBO 6.0 [38] and plotted using Jmol software [39].

## 4. Conclusions

In summary, a novel NHC*-Au(I)-cyanide complex (**2**), three NHC*-Au(I)-dithiocarbamates (**3**–**5**), three NHC*-Au(I)-*p*-mercaptobenzoates (**12**–**14**), and three NHC*-Au(I)-NAC (**15**–**17**) complexes, were synthesised and characterised.

Complexes **2**–**5** and **12**–**17** were based on the NHC* ligand system, as NHCs have been shown to be stronger σ-donors than phosphines. DFT calculations, carried out at the MN15/6-311++G(2df,p)/LANL2TZ(f) level, show the formation of NHC*-AuCl is more desired than the phosphine alternative, Ph_3_P-AuCl. A more negative ΔH and concurrent NBO analysis favours the NHC* ligand. Furthermore, calculated Au–Cl bond distances reveal the bond is shorter in the NHC*-AuCl compound, and therefore, stronger than in the phosphine compound.

Cytotoxicity studies conducted against the human colon carcinoma cell lines HCT-116^wt^, its p53 knockout mutant HCT-116^−/−^, and the *mdr* human breast cancer cell line MCF-7^topo^, show low micromolar and even nanomolar activity. Complex 3 exhibited the best activity with IC_50_ values of 1.5 ± 0.1 μM and 0.28 ± 0.03 μM, against HCT-116^wt^ and MCF-7^topo^ cell lines, respectively. Overall, the NHC*-Au(I)-thiolates proved to be more biologically active than complex **1** or **2**, which lack the influential Au–S bond.

Furthermore, the series of complexes with the NAC derivative (**15**–**17**) were the most successful series of compounds tested. Complexes **15**–**17** displayed consistently high cytotoxic activity when compared to the other sets, strongly suggesting the benefit of conjugating the NHC*-Au(I) to a biological vector. These encouraging results may be valuable in the development of new anticancer drugs that incorporate amino acid derivatives.

## Figures and Tables

**Table 1:** Crystal data and structure refinement for complexes **2**, **12**, **13**, and **14**.

	2	12	13	14
Empirical Formula	C_30_H_24_AuN_3_	C_36_H_29_N_2_O_2_SAu	C_37_H_31_N_2_O_2_SAu	C_38_H_33_N_2_O_2_SAu
Formula Weight (g·mol^−1^)	623.49	750.54	764.66	778.69
Temperature (K)	100(2)	100(2)	100(2)	100(2)
Crystal system	Monoclinic	Triclinic	Triclinic	Monoclinic
Space group	P2_1/m_ (#11)	P$\bar{1}$ (#2)	P$\bar{1}$ (#2)	C2/c (#15)
Unit cell dimensions				
a (Å)	12.8150(7)	9.3234(3)	8.89830(6)	26.1234(3)
b (Å)	6.4797(3)	10.4210(3)	12.12378(8)	10.2154(1)
c (Å)	15.6802(8)	16.0388(5)	15.5000(1)	23.9038(3)
α (°)	90	75.663(3)	103.1637(6)	90
β (°)	112.268(6)	85.553(2)	105.3420(6)	100.387(1)
γ (°)	90	83.264(3)	98.8340(6)	90
Volume (Å^3^)	1204.94(12)	1497.46(8)	1528.942(19)	6274.45(12)
Z	2	2	2	8
Density (calcd) (mg/m^3^)	1.718	1.665	1.661	1.649
Absorption coefficient (mm^−1^)	11.641	5.018	9.964	9.723
F (000)	608	740	756	3088
Crystal size (mm^3^)	0.255 × 0.034 × 0.026	0.194 × 0.121 × 0.082	0.248 × 0.193 × 0.120	0.113 × 0.035 × 0.010
θ (°)	3.727 to 77.196	2.90 to 29.59	3.845 to 76.876	3.44 to 76.91
Index ranges	−16 ≤ h ≤ 15	−12 ≤ h ≤ 12	−11 ≤ h ≤ 11	−32 ≤ h ≤ 32
	−8 ≤ k ≤ 8	−13 ≤ k ≤ 13	−15 ≤ k ≤ 15	−12 ≤ k ≤ 12
	−19 ≤ l ≤ 19	−21 ≤ l ≤ 21	−19 ≤ l ≤ 19	−30 ≤ l ≤ 28
Reflections collected	24,420	20,276	34,268	39,269
Independent reflections R_int_	2760(0.1335)	7306(0.0341)	6409(0.0268)	6584(0.0339)
Completeness to θ_max_ (%)	99.8	99.2	100.0	99.4
Absorption correction	Gaussian	Gaussian	Gaussian	Gaussian
Max and min transmission	0.788 and 0.291	0.714 and 0.472	0.455 and 0.227	0.913 and 0.516
Refinement method	Full-matrix Least-squares on F^2^	Full-matrix Least-squares on F^2^	Full-matrix Least-squares on F^2^	Full-matrix Least-squares on F^2^
Data/restraints/parameters	2760/0/147	7306/0/380	6409/0/389	6584/0/398
Goodness-of-fit on F2	1.139	1.046	1.085	1.041
Final R indices [I > 2σ(I)]	R1 = 0.0442, wR2 = 0.0983	R1 = 0.0255, wR2 = 0.0437	R1 = 0.0178, wR2 = 0.0439	R1 = 0.0266, wR2 = 0.0674
R indices (all data)	R1 = 0.0464, wR2 = 0.0998	R1 = 0.0324, wR2 = 0.0465	R1 = 0.0187, wR2 = 0.0442	R1 = 0.0305, wR2 = 0.0701
Largest diff. peak and hole	1.982 and −1.411	1.009 and −0.762	0.639 and −0.859	1.508 and −1.500

**Table 2:** Selected bond angles for **2**, **12**, **13**, and **14**.

	2	12	13	14
Au–C(8)	2.031(8)	2.012(3)	2.008(2)	2.008(3)
Au–C(30)	2.026(9)			
C(30)–N(3)	1.113(12)			
Au–S(1)		2.2856(7)	2.2851(6)	2.3012(8)
S(1)–C(30)		1.751(3)	1.755(2)	1.735(3)
C(36)–O(1)		1.249(3)	1.211(3)	1.215(5)
C(36)–O(2)		1.304(3)	1.349(3)	1.337(5)
O(2)–C(37)			1.442(3)	1.450(5)

Bond lengths (Å).

**Table 3:** Selected bond angles for **2**, **12**, **13**, and **14**.

	2	12	13	14
C(8)–Au–S		177.48(8)	175.20(6)	173.45(9)
C(8)–Au–C(30)	179.6(4)			
Au–S–C(30)		108.40(10)	109.44(8)	108.83(12)
Au–C(30)–N(3)	177.0(8)			
S(1)–C(30)–S(2)				
O(1)–C(36)–O(2)		122.8(3)	122.9(2)	123.4(3)
O(1)–C(36)–C(33)		120.8(3)	125.2(2)	124.2(4)
O(2)–C(36)–C(33)		116.4(3)	111.9(2)	112.4(3)
C(36)–O(2)–C(37)			115.4(2)	117.0(3)

Bond angle (°).

**Table 4:** IC_50_ values (μM) of compounds **2, 3**, and **12**–**17** against MCF-7^topo^, HCT-116^wt^, and HCT-116 p53^−/−^ cells after 72 h of incubation.

	HCT-116^wt^	HCT-116 p53^−/−^	MCF-7^topo^
**2**	14.8 ± 1.9	-	10.8 ± 0.9
**3**	1.5 ± 0.1	-	0.28 ± 0.03
**4**	8.0 ± 0.1	3.8 ± 0.4	0.36 ± 0.03
**5**	6.2 ± 0.3	2.0 ± 0.6	1.5 ± 0.3
**12**	5.5 ± 0.1	2.7 ± 0.2	5.4 ± 0.5
**13**	18.1 ± 6.5	9.5 ± 0.6	21.3 ± 3.4
**14**	6.8 ± 0.2	7.9 ± 0.2	13.2 ± 3.7
**15**	4.5 ± 1.2	6.6 ± 0.3	7.1 ± 0.3
**16**	2.8 ± 0.1	4.5 ± 0.6	6.3 ± 0.5
**17**	2.9 ± 0.1	3.7 ± 0.2	5.4 ± 0.5

## Supplementary Materials

See attached for ^1^H and ^13^C-NMR spectra of all novel compounds.
