# Supplementary material for: Synthesis and Cytotoxicity Studies of Novel NHC*-Gold(I) Complexes Derived from Lepidiline A
Source: Molecules. 2018 Aug 14;23(8):2031. doi: 10.3390/molecules23082031 (PMC6222482; doi:10.3390/molecules23082031)
Supplement: Supplementary file 1 [file molecules-23-02031-s001.pdf]

Supplementary Materials for

Synthesis and Cytotoxicity Studies of Novel NHC*-Gold(I) Complexes Derived from Lepidiline A

Danielle Curran [1], Oyinlola Dada [1], Helge Müller-Bunz [1], Matthias Rothemund [2], Goar Sánchez-Sanz [1,3], Rainer Schobert [2], Xiangming Zhu [1] and Matthias Tacke [1,*]

[1] School of Chemistry, University College Dublin, Belfield, Dublin 4, Ireland
[2] Organic Chemistry Laboratory, University of Bayreuth, Universitätsstr. 30, 95440 Bayreuth, Germany
[3] Irish Centre of High-End Computing, Grand Canal Quay, Dublin 2, Ireland
* Correspondence: matthias.tacke@ucd.ie

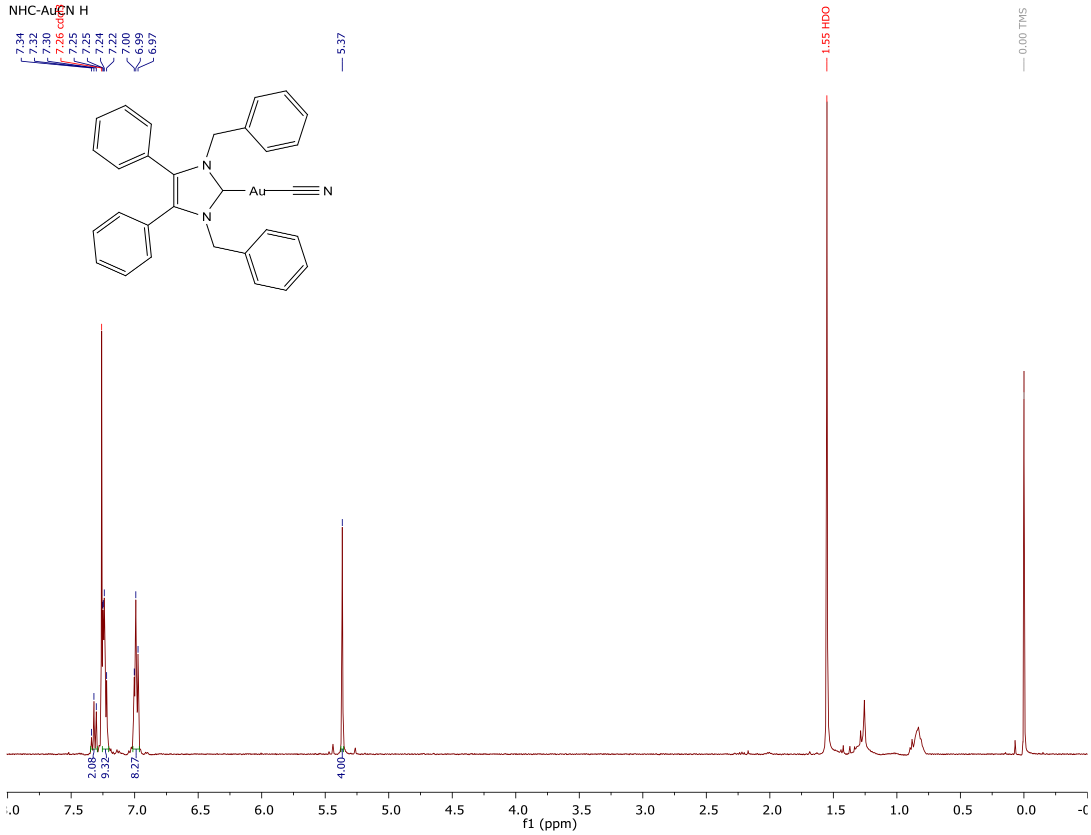

S 1. ^{1}H NMR (400 MHz, CDCl3) spectrum of the new compound **2**.

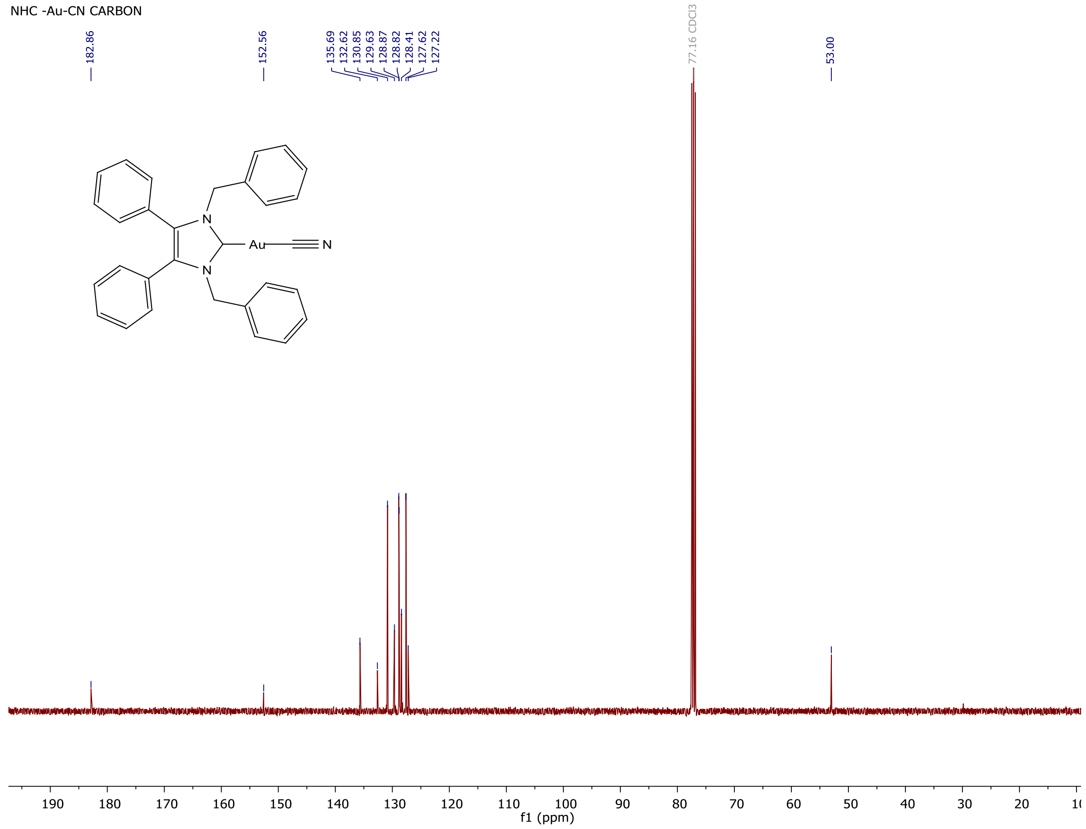

S 2. ^{13}C NMR (101 MHz, CDCl3) spectrum of the new compound **2**.

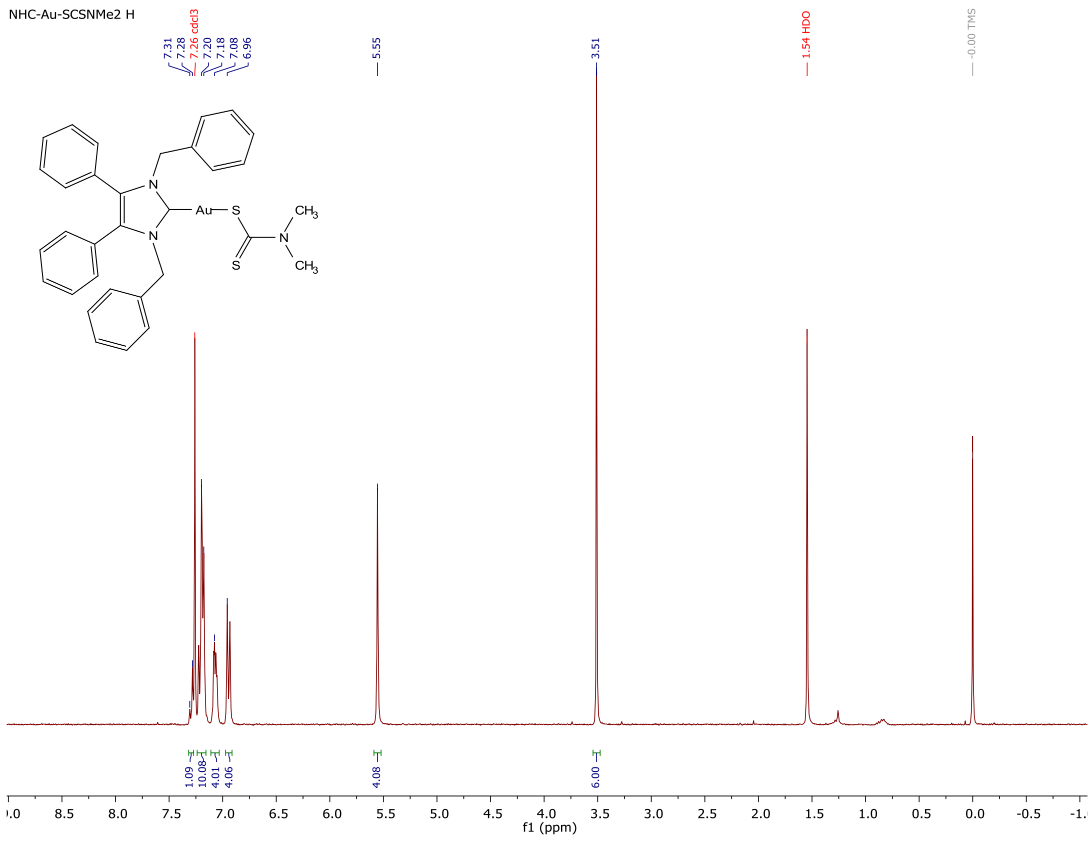

S 3. ^{1}H NMR (300 MHz, CDCl3) spectrum of the new compound **3**.

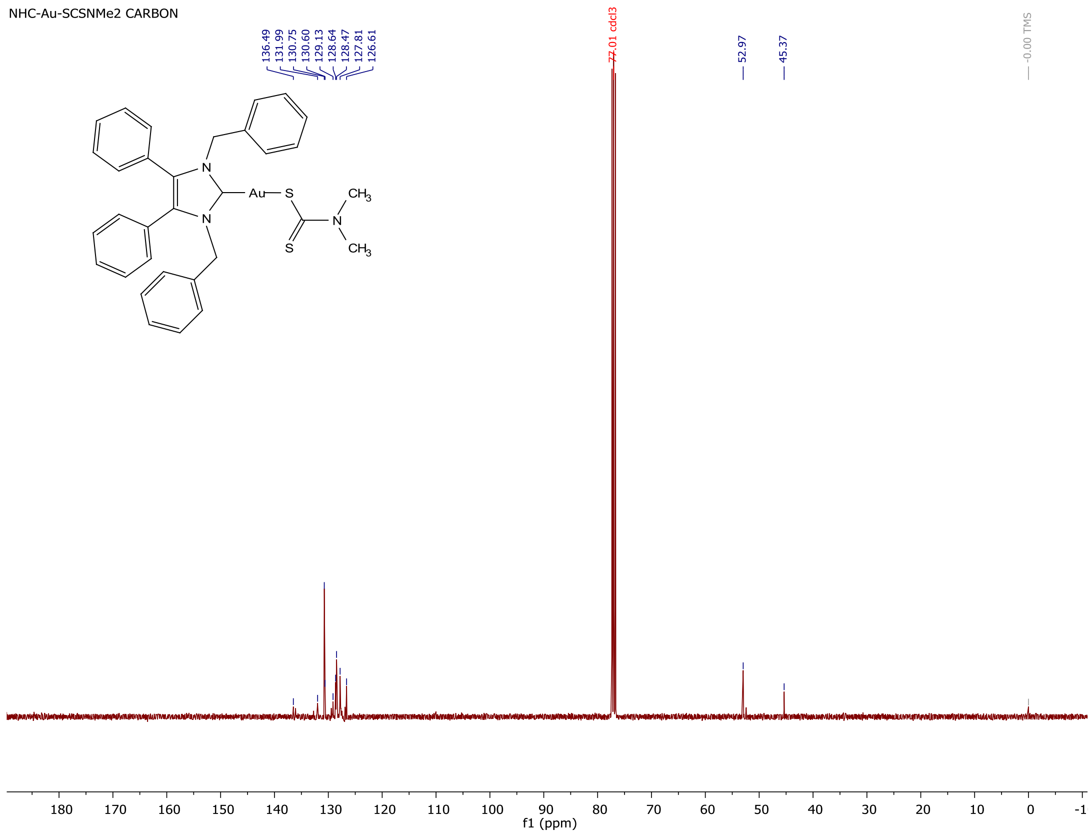

S 4. ^{13}C NMR (101 MHz, CDCl3) spectrum of the new compound **3**.

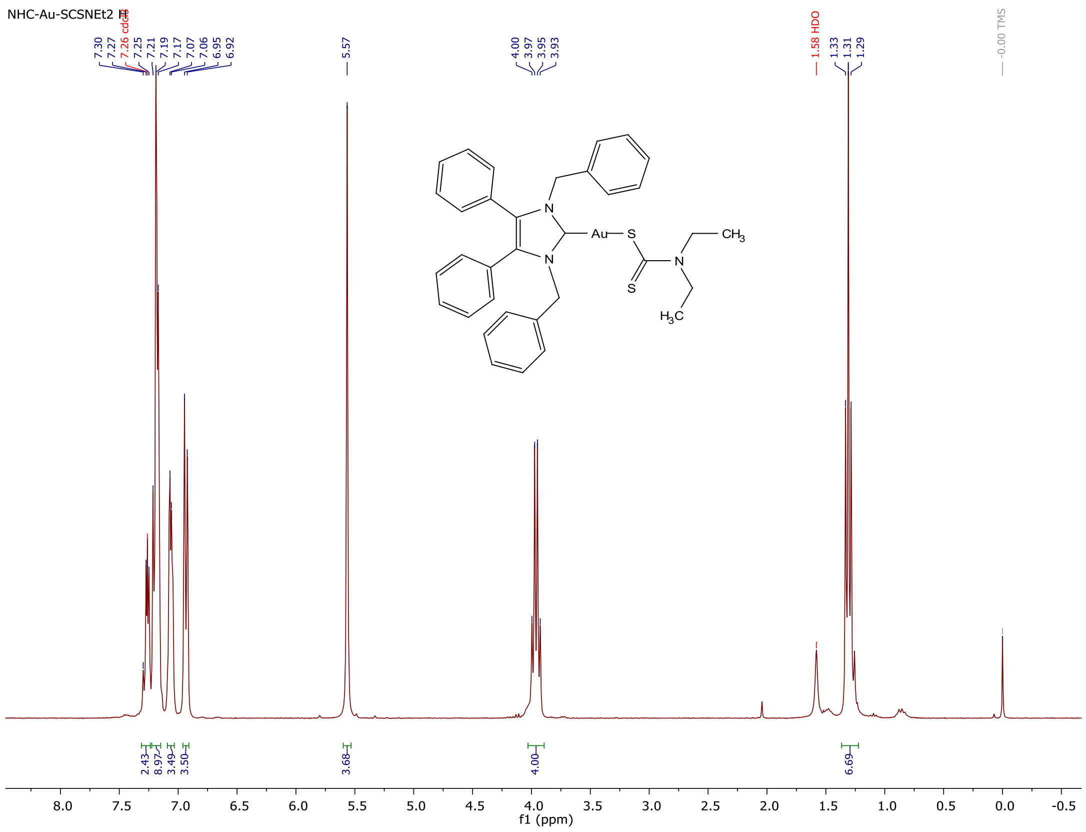

S 5. ^{1}H NMR (300 MHz, CDCl3) spectrum of the new compound **4**.

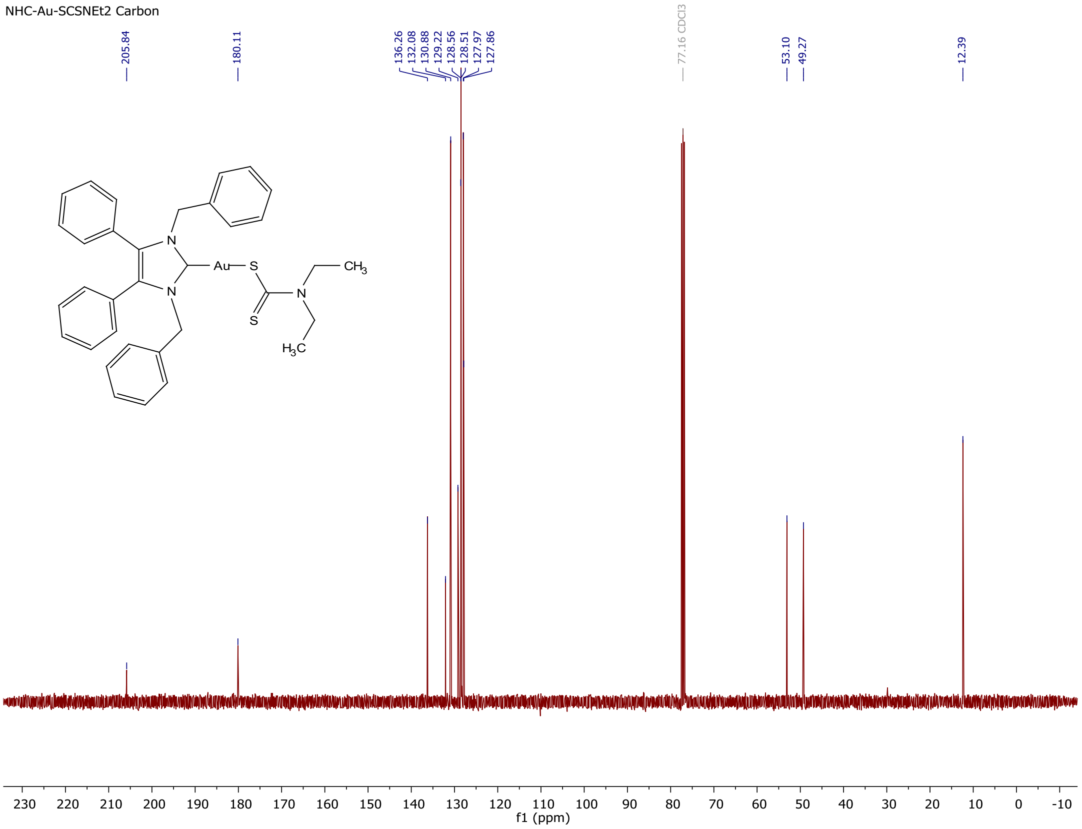

S 6. ^{13}C NMR (101 MHz, CDCl3) spectrum of the new compound **4**.

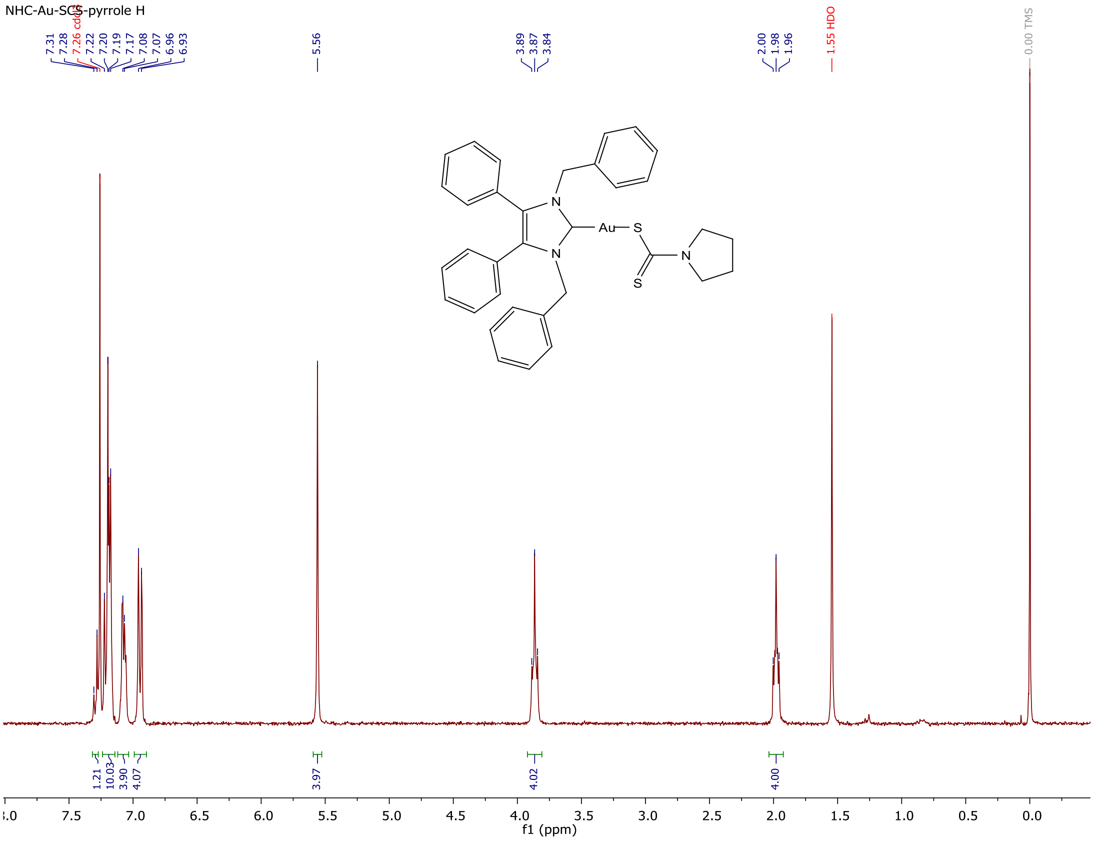

S 7. ^{1}H NMR (300 MHz, CDCl3) spectrum of the new compound **5**.

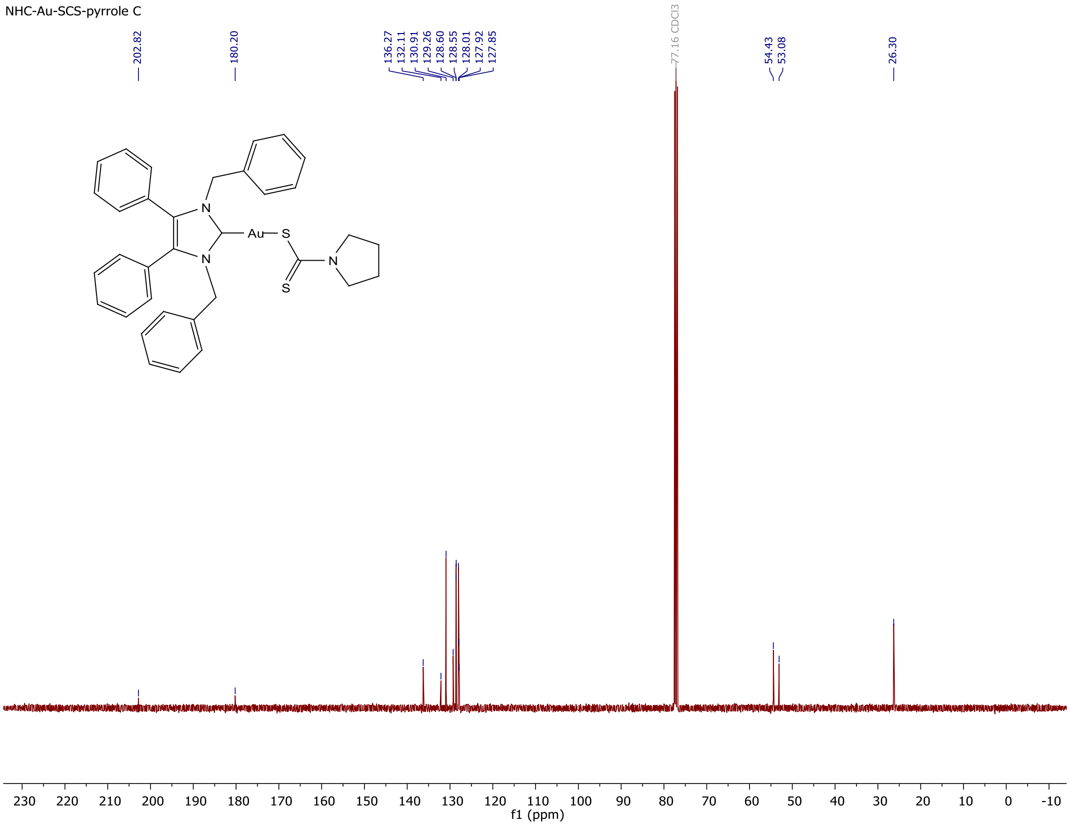

S 8. ^{13}C NMR (101 MHz, CDCl3) spectrum of the new compound **5**.

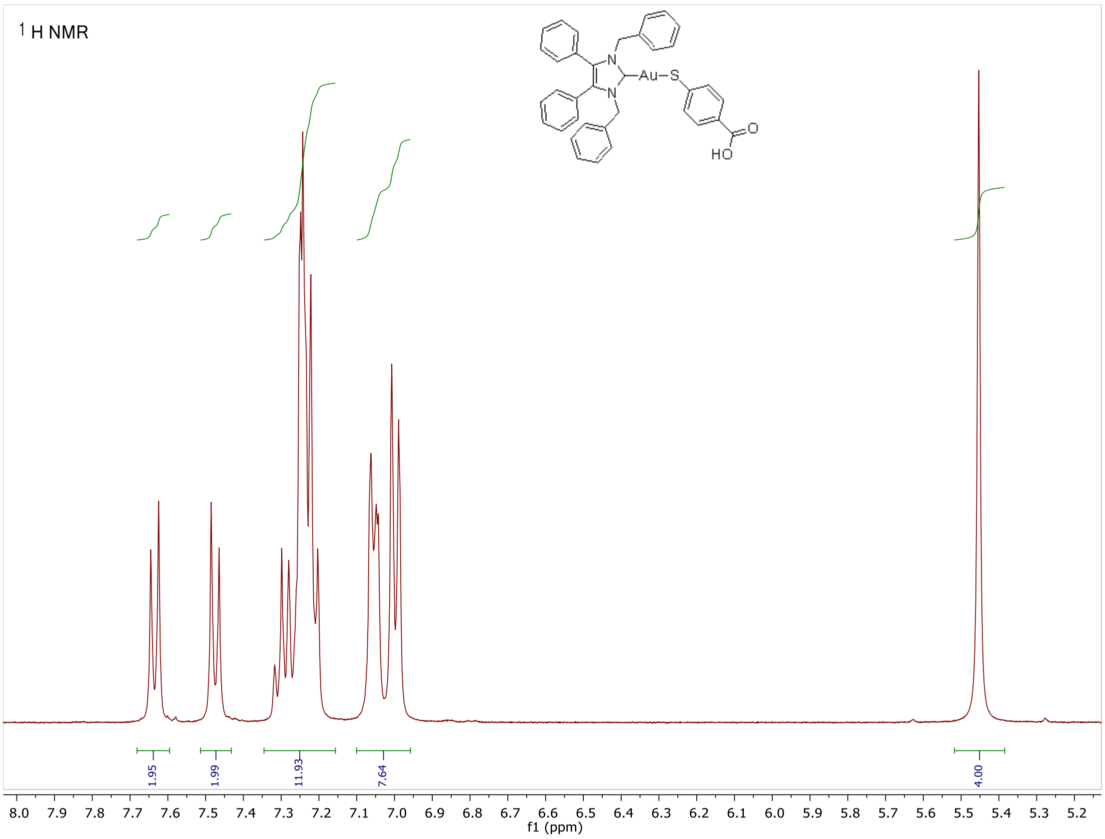

S 9. ^{1}H NMR (400 MHz, CDCl3) spectrum of the new compound **12**.

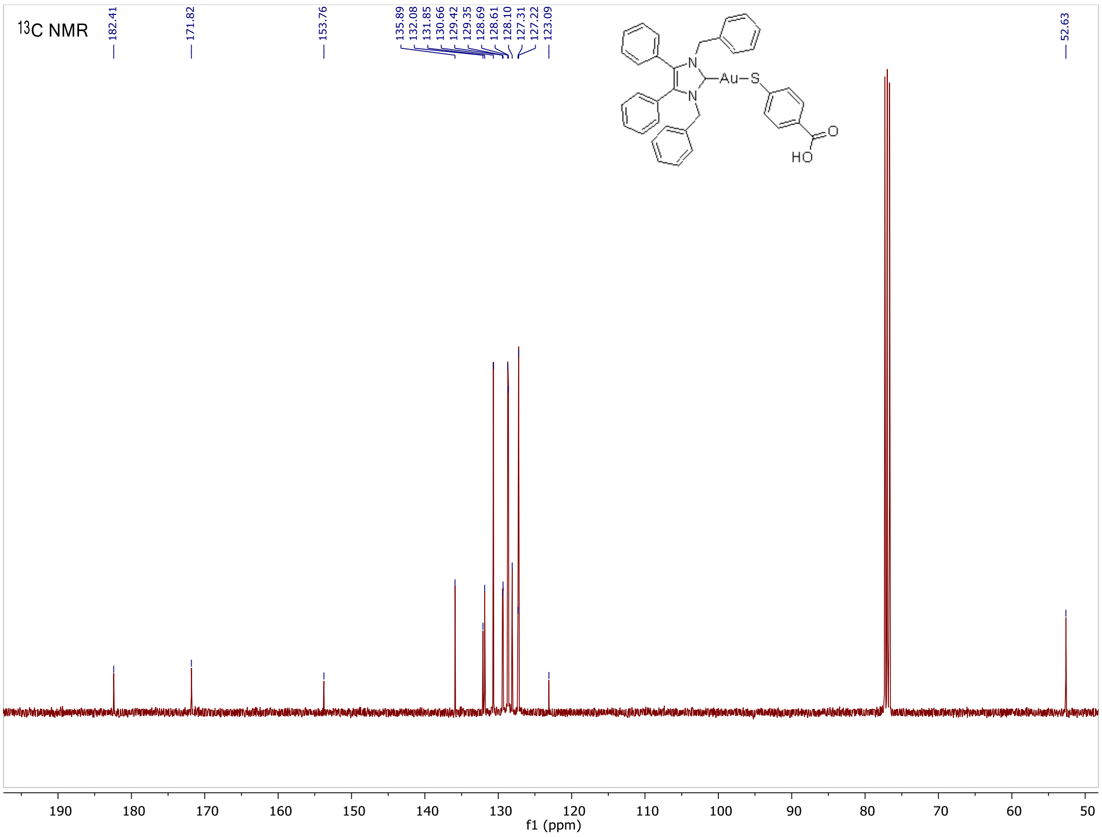

S 10. ^{13}C NMR (101 MHz, CDCl3) spectrum of the new compound **12**.

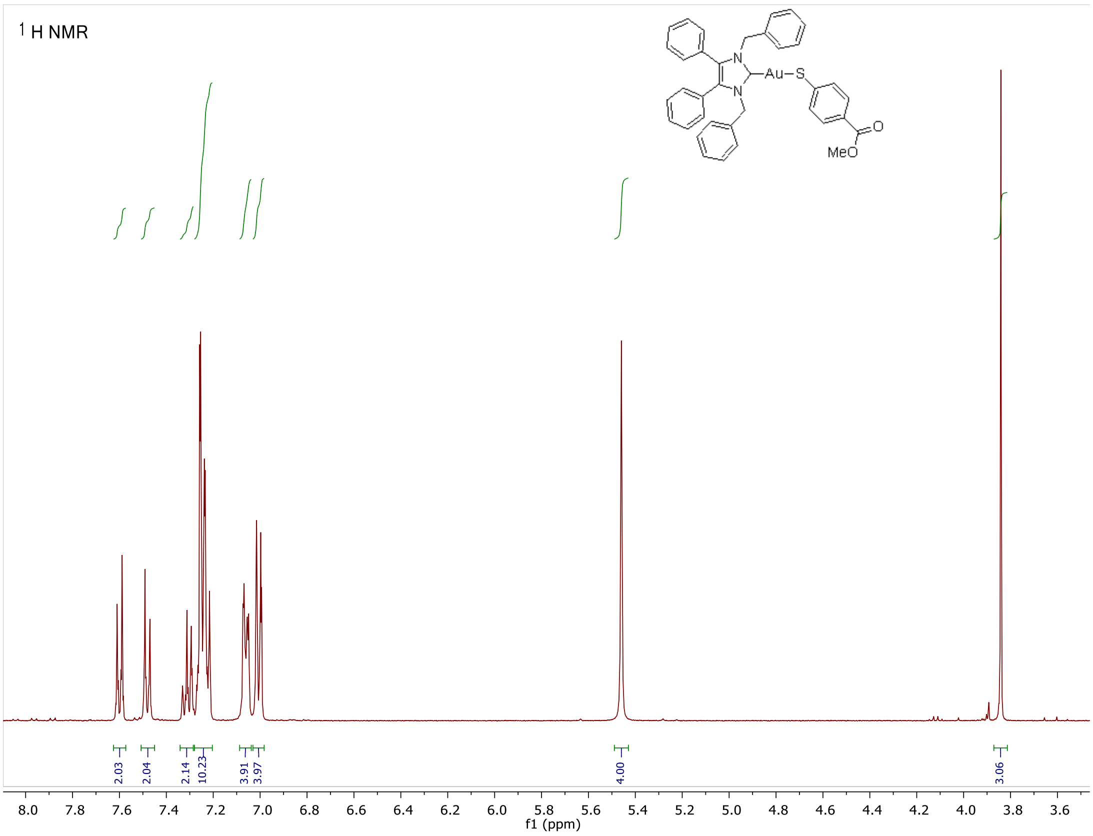

S 11. ^{1}H NMR (400 MHz, CDCl3) spectrum of the new compound **13**.

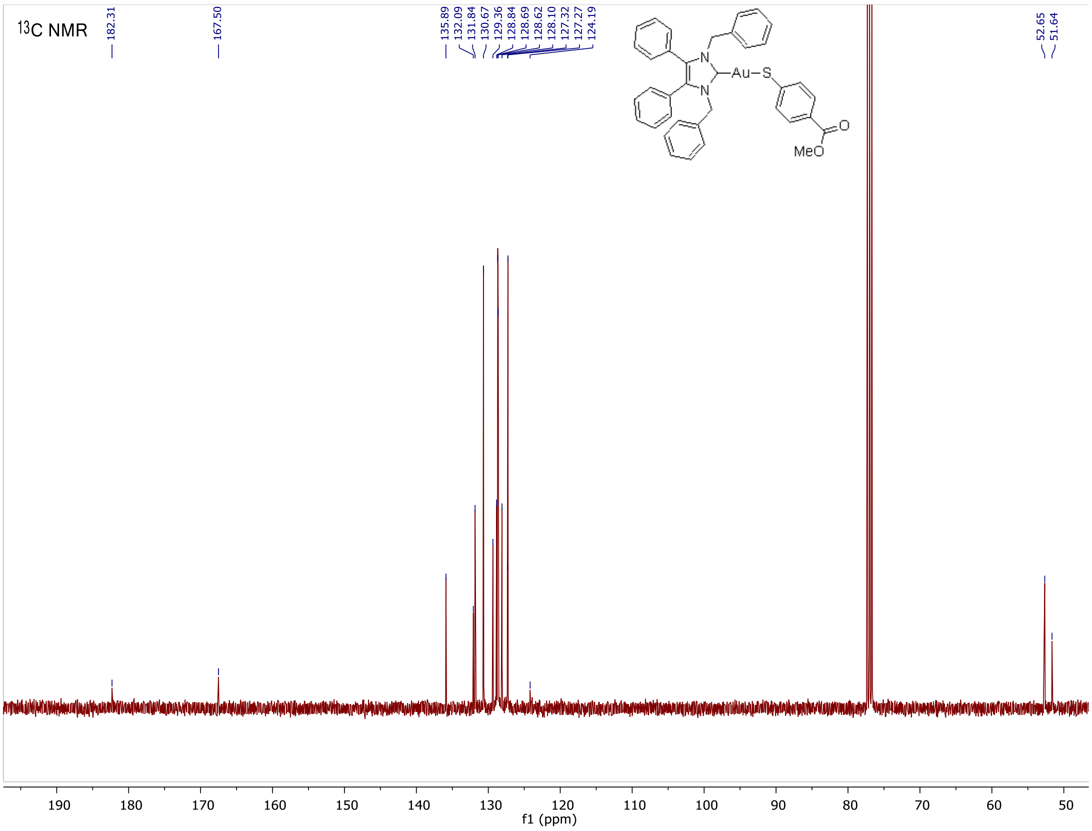

S 12. ^{13}C NMR (101 MHz, CDCl3) spectrum of the new compound **13**.

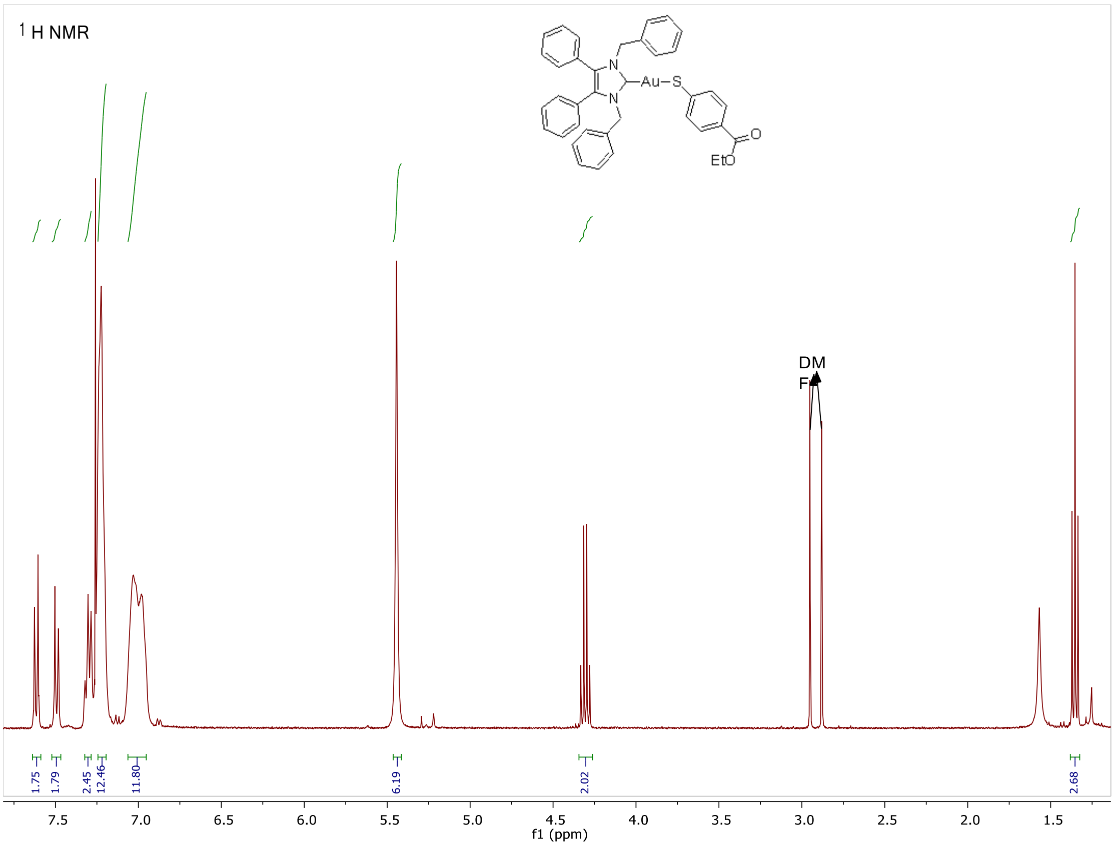

S 13. ^{1}H NMR (300 MHz, CDCl3) spectrum of the new compound **14.**

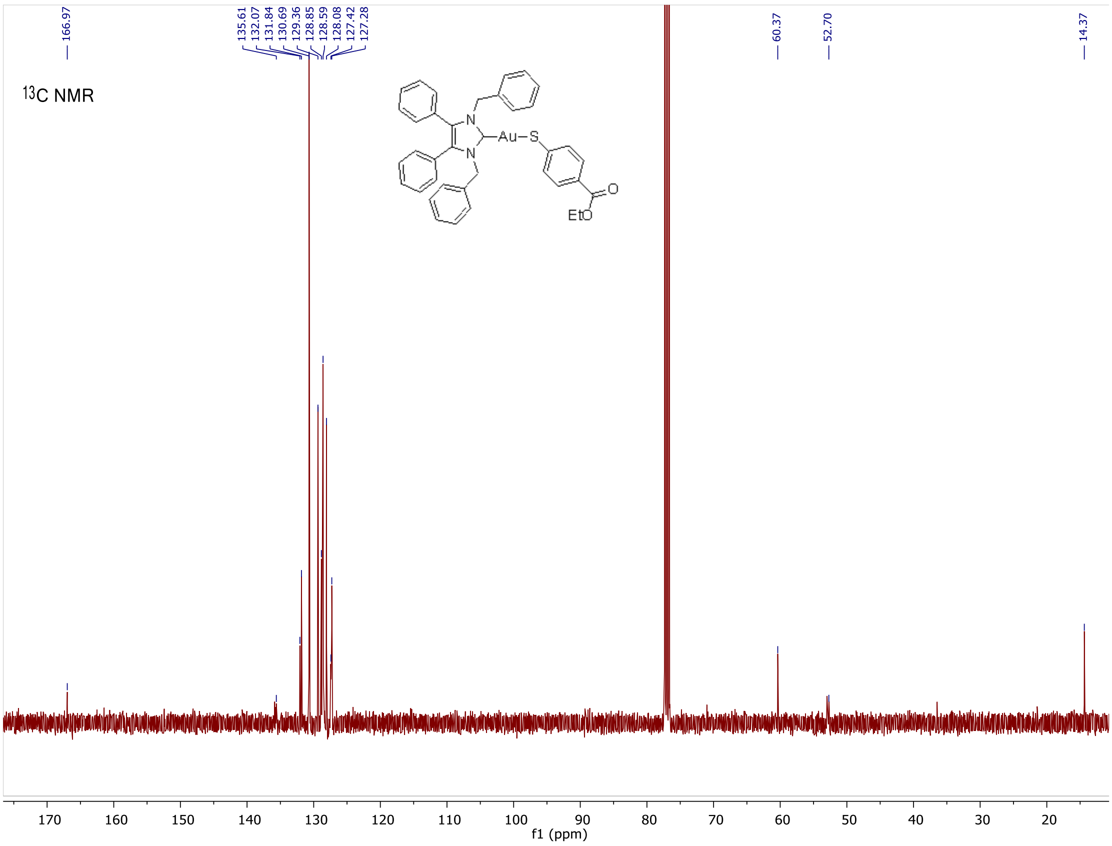

S 14. ^{13}C NMR (101 MHz, CDCl3) spectrum of the new compound **14**.

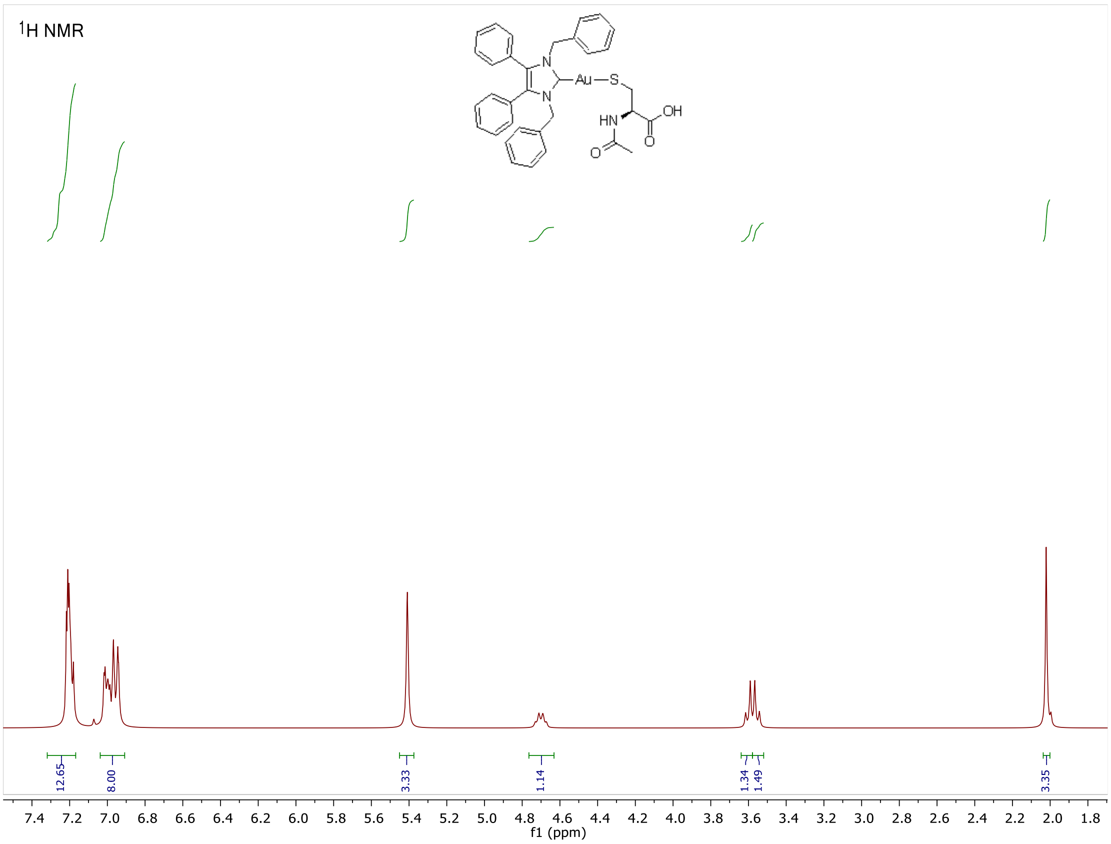

S 15. ^{1}H NMR (300 MHz, CDCl3) spectrum of the new compound **15**.

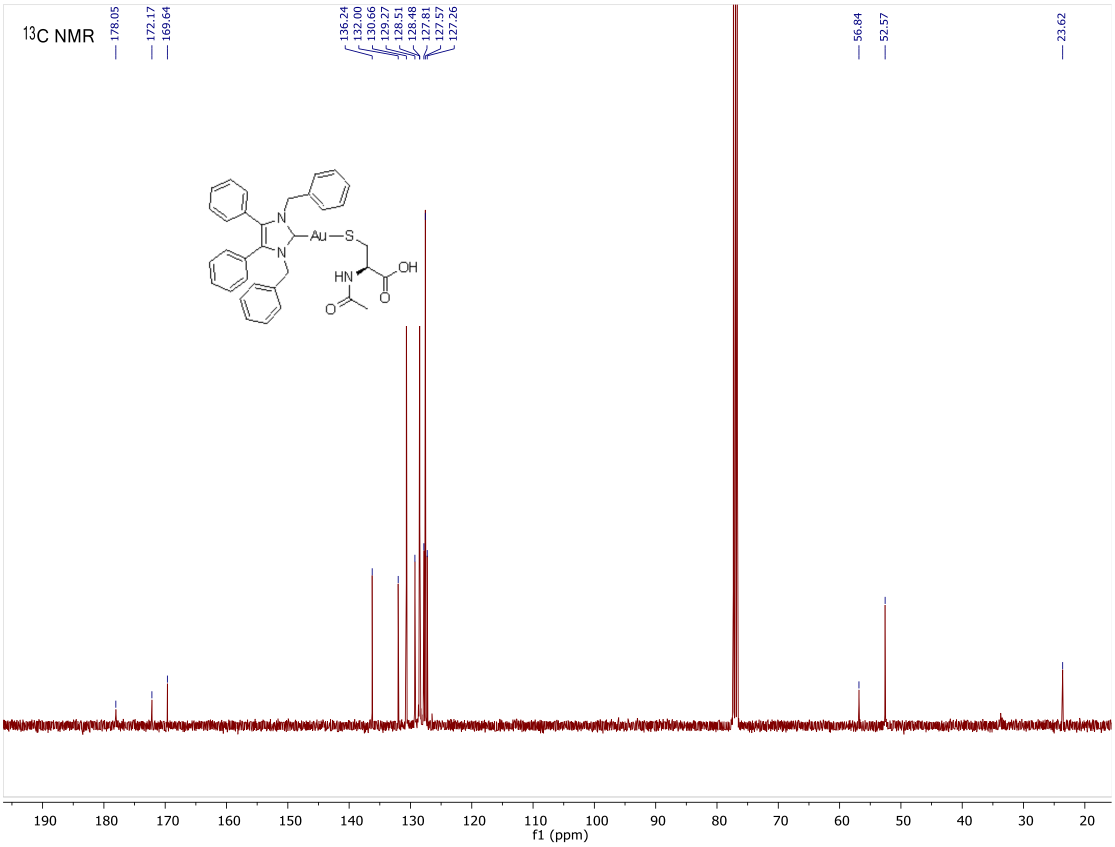

S 16. ^{13}C NMR (101 MHz, CDCl3) spectrum of the new compound **15**.

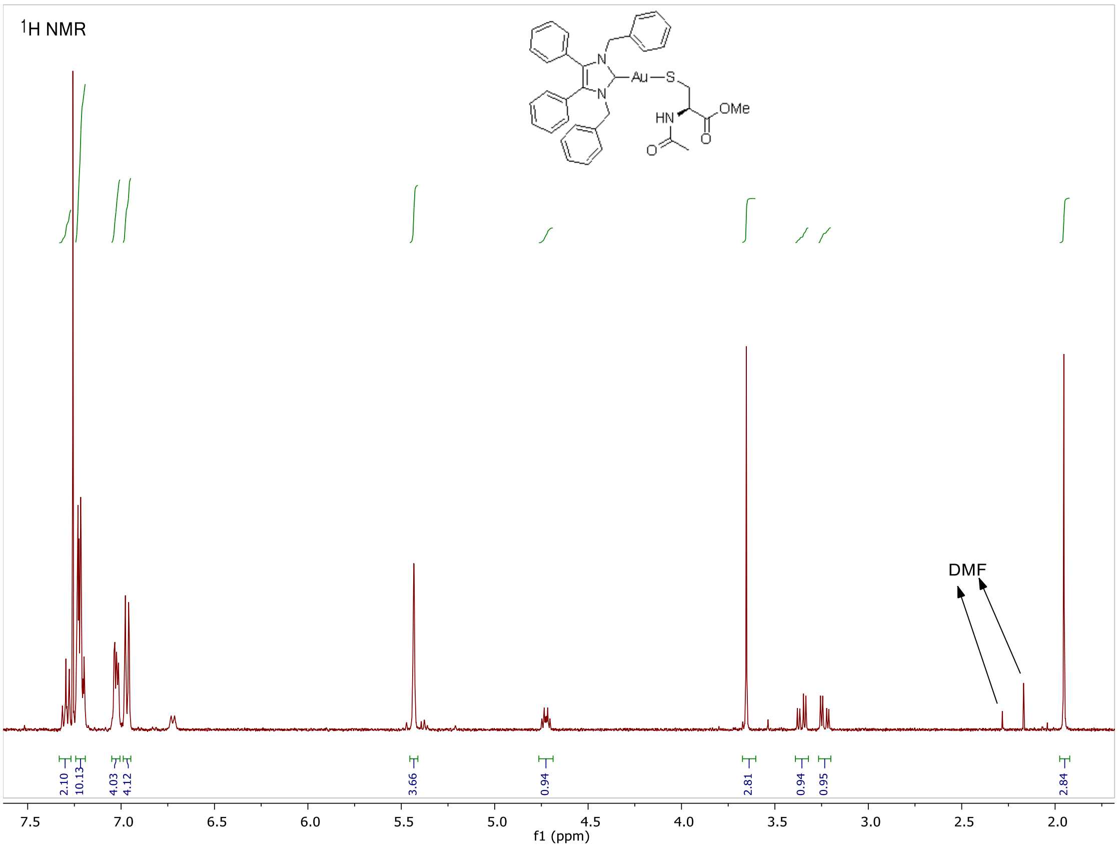

S 17. ^{1}H NMR (300 MHz, CDCl3) spectrum of the new compound **16**.

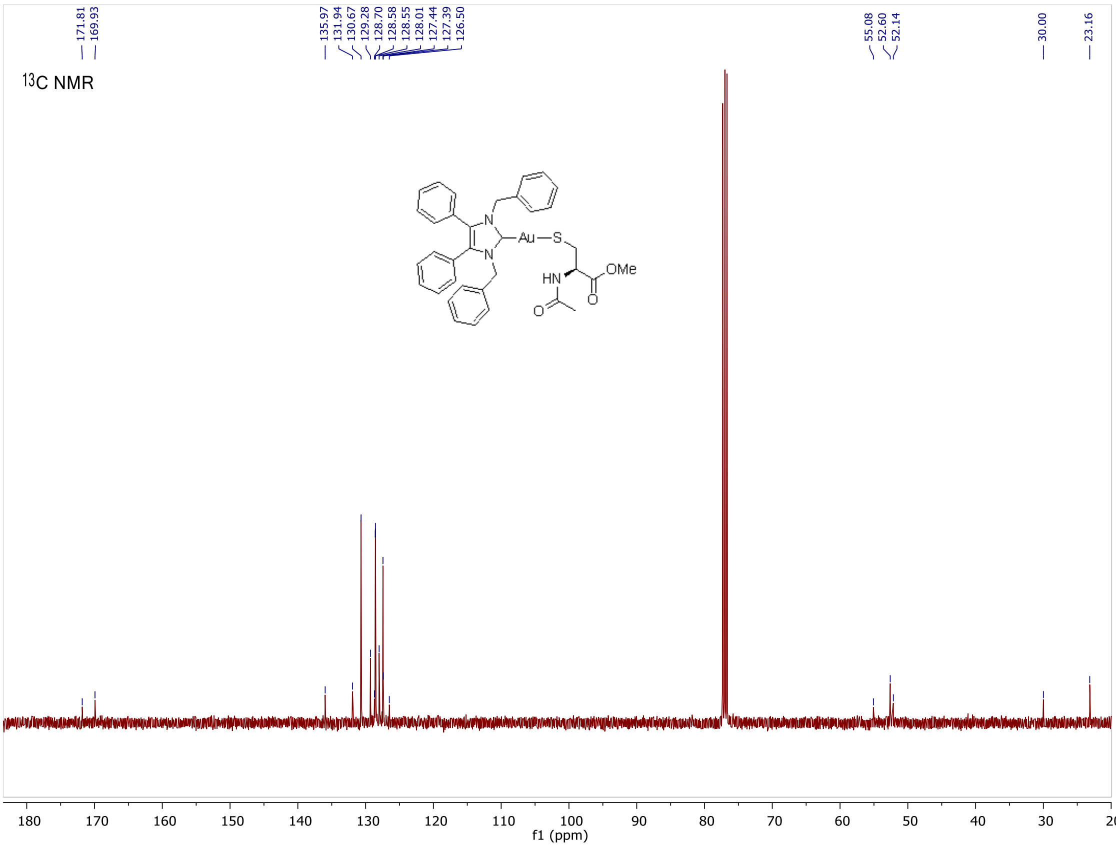

S 18. ^{13}C NMR (101 MHz, CDCl3) spectrum of the new compound **16**.

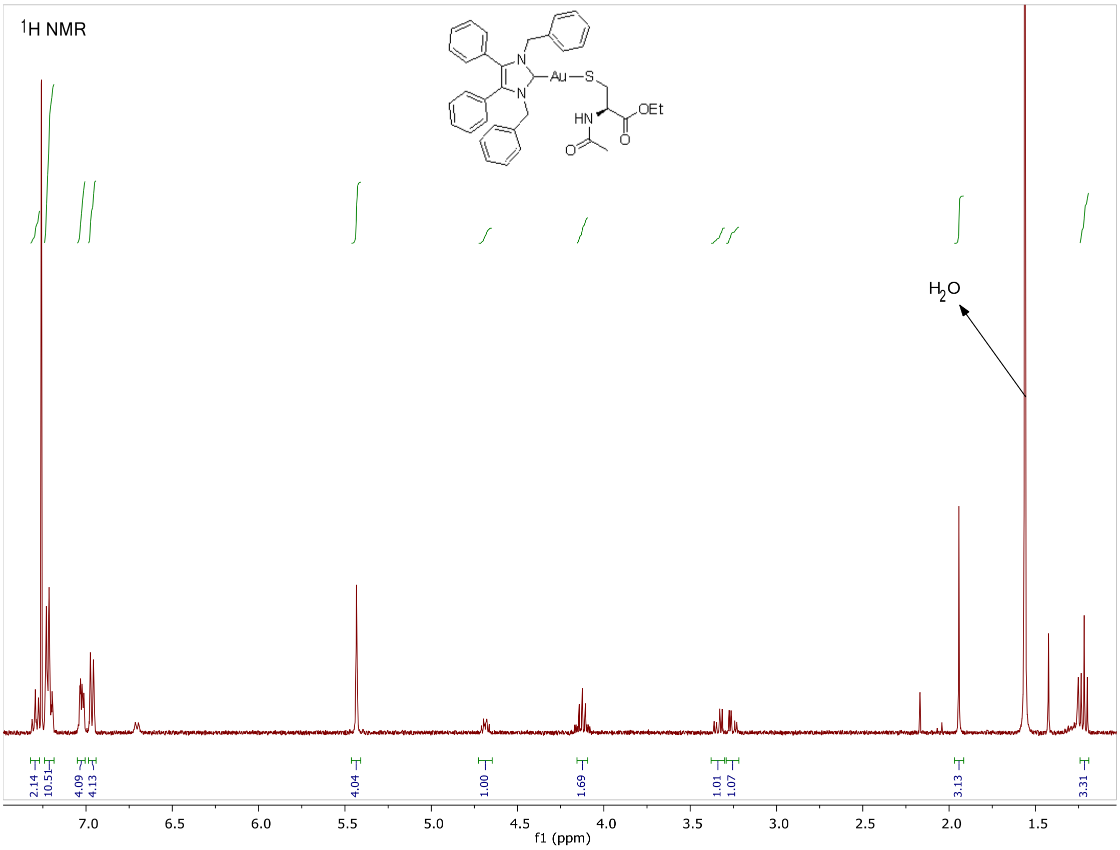

S 19. 1H NMR (400 MHz, CDCl3) spectrum of the new compound **17**.

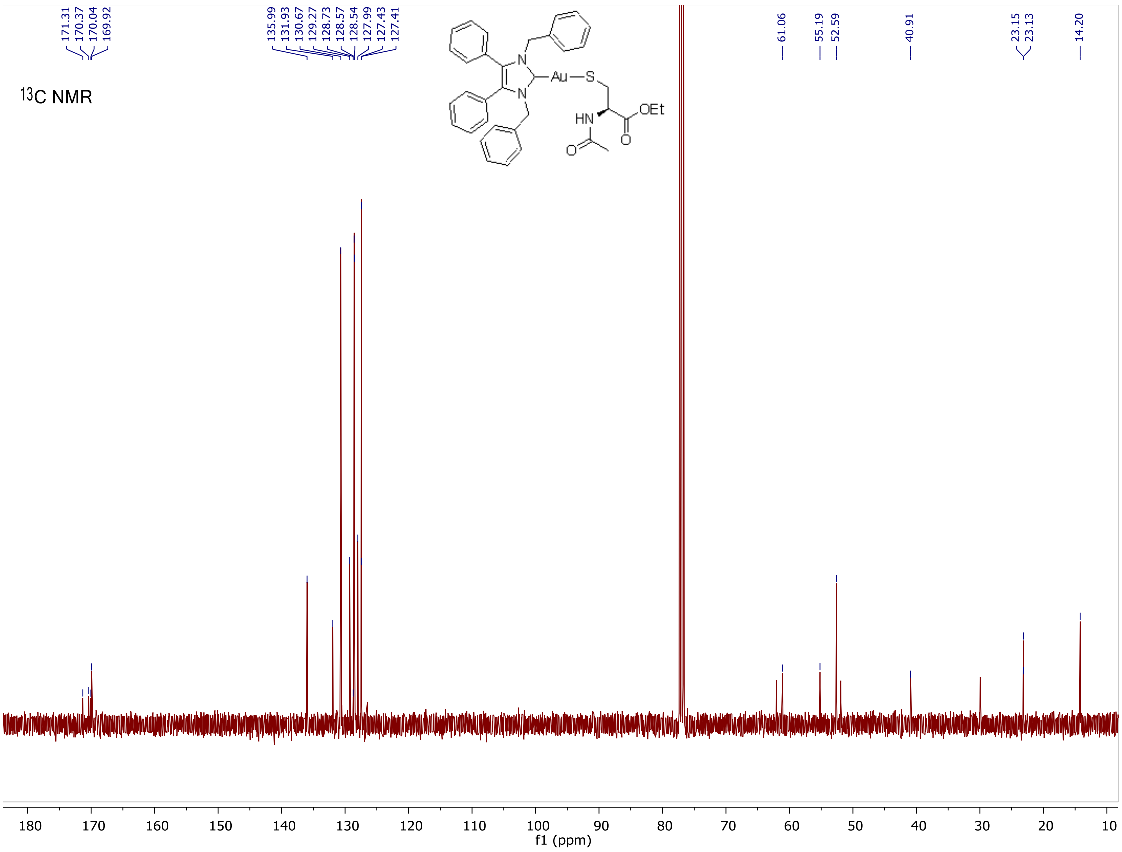

S 20. 13C NMR (101 MHz, $CDCl_3$) spectrum of the new compound **17**.
